# USP7 inhibition perturbs proteostasis and tumorigenesis in triple-negative breast cancer

**DOI:** 10.1038/s41523-026-00974-5

**Published:** 2026-05-23

**Authors:** Ahhyun Kim, Priya Gopalakrishnan, Marina Suárez-Pizarro, Claire C. Chen, Xianxi Wang, Sydney M. McCoy, Nikita Umesh, Angie Mordant, Natalie K. Barker, Laura E. Herring, Rasha T. Kakati, Philip M. Spanheimer, Michael J. Emanuele, Claudia A. Benavente

**Affiliations:** 1 Department of Pharmaceutical Sciences, University of California, Irvine, CA USA; 2 Chao Family Comprehensive Cancer Center, University of California, Irvine, CA USA; 3 Department of Pharmacology, The University of North Carolina, Chapel Hill, NC USA; 4 Lineberger Comprehensive Cancer Center, The University of North Carolina, Chapel Hill, NC USA; 5 Department of Developmental and Cell Biology, University of California, Irvine, CA USA; 6 UNC Metabolomics and Proteomics Core Facility, The University of North Carolina, Chapel Hill, NC USA; 7 Department of Surgery, Department of Genetics, The University of North Carolina, Chapel Hill, NC USA

**Keywords:** Breast cancer, Metastasis, Cancer, Targeted therapies, Ubiquitylation

## Abstract

The deubiquitinase USP7 is a critical regulator of tumorigenesis, known for stabilizing the MDM2-p53 pathway. Emerging evidence highlights USP7’s p53-independent roles in proliferation and tumorigenesis. Our study reveals that USP7 is broadly upregulated in breast cancer, including triple-negative breast cancer (TNBC), a subtype characterized by near-universal *TP53* loss. This provides a model to investigate p53-independent functions of USP7. Using TNBC models, we define p53-independent roles of USP7 in regulating proteostasis and tumorigenesis. Importantly, genetic and pharmacologic USP7 inactivation impaired tumor progression in TNBC models. To explore USP7’s role in p53-mutant TNBCs, we performed deep quantitative proteomics across TNBC cell lines, identifying shared USP7 targets involved in cell proliferation, genome stability, and proteostasis. Acute USP7 inactivation allowed us to infer proximally controlled proteins that are likely direct targets. Surprisingly, many of the proteins downregulated by USP7 inhibition are E3 ubiquitin ligases. Thus, a key USP7 function in TNBC is to antagonize the degradation of ubiquitinating enzymes, since these enzymes are often susceptible to auto-ubiquitination and degradation. Notably, we identified TOPORS, a dual ubiquitin- and SUMO-ligase, among novel USP7 substrates. TOPORS interacts with the BRCA1-A DNA damage repair complex, suggesting a USP7-TOPORS-BRCA1-A axis that might further explain the continued proliferation of genomically unstable TNBCs. Collectively, these data nominate USP7 as a potential therapeutic vulnerability in TNBC.

## Introduction

Triple-negative breast cancer (TNBC) is defined by the absence of estrogen and progesterone receptors (ER and PR) and the lack of amplification of HER2/ERBB2 receptor tyrosine kinase. TNBCs largely overlap with the basal-like intrinsic molecular breast cancer subtype^1^. This subtype is associated with poor clinical outcomes and remains an independent predictor of mortality despite advances in breast cancer therapy^2,3^. Unlike other breast cancer subtypes, TNBC tumors lack recurrent, targetable oncogenic drivers (e.g., RAS, PI3K) but are characterized by near-universal loss of the *TP53* tumor suppressor, leading to widespread genomic instability^4^. Further, TNBCs exhibit functional loss of the retinoblastoma tumor suppressor (*RB1*), rendering CDK4/6 inhibitors also ineffective for TNBC treatment^4,5^. Current therapeutic options for TNBC remain limited. PARP inhibitors provide benefit in the subset of patients harboring BRCA1 mutations (5-10% of cases), and more recently, immune checkpoint inhibitors in combination with chemotherapy have shown efficacy in both metastatic and early-stage disease for select patients^6–8^. Despite these advances, there remains a significant unmet clinical need for improved therapeutic strategies for TNBC patients.

Ubiquitination is a three-step enzymatic cascade in which E3 ubiquitin ligases modify substrates with the small protein ubiquitin. Polyubiquitination often leads to substrate degradation, making ubiquitin a central regulator in diverse cellular processes and disease states^9^. Ubiquitination regulates multiple cellular functions, including gene expression and cell cycle progression, and can be reversed by catalytic proteases termed deubiquitinases (DUBs)^10^. By removing ubiquitin from substrates, DUBs prevent their proteasomal degradation and often increase the abundance of their targets post-transcriptionally. Among the ~100 identified DUBs, ubiquitin-specific protease 7 (USP7) is the most extensively studied^11^. USP7 stabilizes proteins involved in critical cellular processes, including DNA replication, cell cycle regulation, and apoptosis, and is overexpressed in multiple cancers, where it supports tumor cell survival^12^. Most research on USP7 has centered on its role in regulating TP53 through stabilization of its E3 ligase MDM2^13–16^, though in at least 50% of tumors, *TP53* is inactivated by mutations^17^. Recent studies have identified TP53-independent USP7 substrates, including FOXM1, that contribute to tumorigenesis in TNBC^18^. Given the near-universal loss of TP53 in TNBC, this subtype provides a biologically defined context to investigate p53-independent functions of USP7.

In this study, we investigated the role of USP7 in TNBC progression, focusing on its impact on tumor growth, metastasis, and downstream regulatory networks. Leveraging TNBC as a model system characterized by near-universal TP53 loss, we specifically sought to define p53-independent functions of USP7. Using complementary genetic and pharmacological approaches, we examined how USP7 inactivation affects tumor behavior in vitro and in vivo. Through integrative proteomic and transcriptomic analyses, we identified key pathways and substrates regulated by USP7, including proteins involved in ubiquitination and DNA repair. Our findings define a p53-independent role for USP7 in coordinating proteostasis and tumor progression, and support further investigation of USP7 as a potential therapeutic target across tumor contexts.

## Results

### Recurrent USP7 amplification and overexpression are associated with poor clinical features in breast cancer

**Fig. 1:**
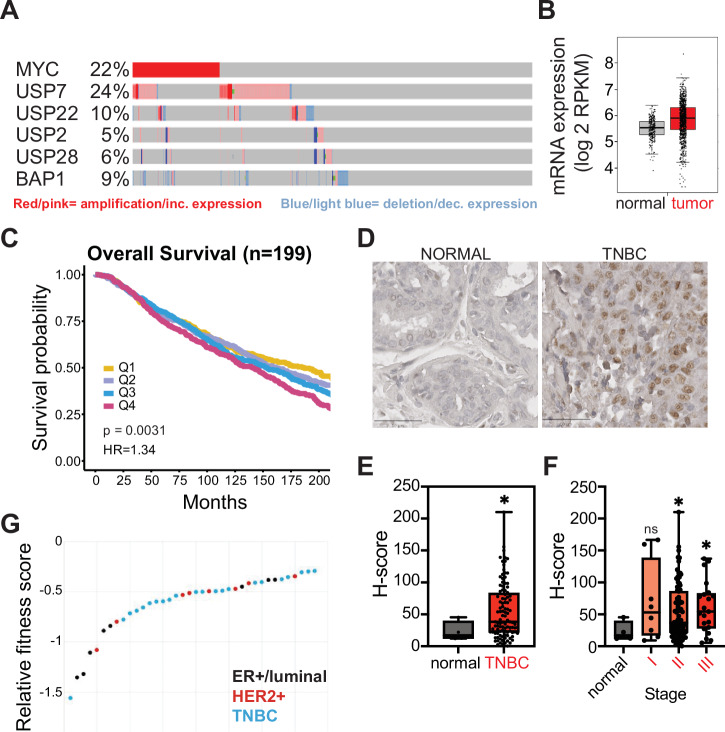
USP7 overexpression is associated with poor prognosis in breast cancer. TCGA Firehose Legacy data for breast cancer tumors shows **A** USP7 is recurrently amplified and overexpressed in breast cancer. Red = amplification; pink = increased expression; blue = deletion; light blue = decreased expression; and **B** USP7 mRNA expression is elevated across all breast cancer tumors (*n* = 1085, red) compared to normal breast tissue (*n* = 291, gray). **C** Overall survival analysis from the METABRIC breast cancer database shows that high USP7 expression correlates with poorer survival (*n* = 199). Patients grouped into quartiles based on USP7 expression: Q1 (yellow) = lowest USP7 expression; Q4 (magenta)=highest USP7 expression. Log-rank test *p* = 0.0031, HR = 1.34. **D** Representative IHC images of core human tissues from normal breast and TNBC tissue microarray stained for USP7. Scale bar = 50 μm. **E** Boxplot for H-scores for USP7 protein expression in normal (*n* = 4) and TNBC (*n* = 103) tissue. The box extends from the 25th to the 75th percentiles, and the median is in between the box. Whiskers show min and max with all samples shown as dots. *p = 0.0385 by one-tailed Mann–Whitney test. **F** Boxplot for H-scores for USP7 protein expression in normal (*n* = 4) and stage I (*n* = 8; *p* = 0.1071), stage II (*n* = 76; *p* = 0.0585), and stage III (*n* = 19; *p* = 0.0106) TNBC tissue. ns = not significant, *p < 0.1 by one-tailed Mann–Whitney test. **G** Analysis of DepMap data on the effect of USP7 CRISPR KO on breast cancer cell fitness/proliferation separated by pathologic breast cancer subtypes.

Elevated levels of USP7 have been observed in many cancers. Analysis of breast cancer patient data from 1108 tumors analyzed by RNA sequencing through TCGA (Firehose Legacy, accessed via cBioPortal) and compared to diploid controls indicates that *USP7* is recurrently amplified at the chromosomal level and significantly overexpressed (Fig. 1A, B). USP7 is among the most recurrently upregulated DUBs in breast cancer, with genomic amplification or mRNA overexpression observed in approximately 24% of tumors, a rate higher than the well-known oncogene *MYC* (22%), as well as other cancer associated DUBs, including USP22, USP2, USP28 and BAP1 (Fig. 1A). Importantly, *USP7* overexpression was observed across multiple breast cancer subtypes, indicating that its dysregulation is not subtype-restricted (Supplemental Fig. 1).

Given the near-universal loss of *TP53* in TNBC, we therefore leveraged this subtype as a biologically defined context to examine the clinical and functional consequences of USP7 expression. In a TNBC subset from METABRIC^19,20^ (accessed via cBioPortal), high USP7 expression (top quartile, Q4) was associated with worse overall survival compared to patients with low USP7 expression (lowest quartile, Q1) in univariate analysis (*n* = 199; Fig. 1C). However, in multivariate Cox regression adjusting for PAM50, age at diagnosis, tumor stage, and intrinsic molecular subtype, USP7 expression was not independently associated with survival (HR 1.04 95% CI 0.97–1.13, *p* = 0.25). These findings indicate that USP7 is broadly overexpressed in breast cancer, including TNBC, and is associated with poor clinical features, but not as an independent prognostic factor.

Since proteins in the ubiquitin pathway are often regulated post-translationally, we examined USP7 protein levels. Immunohistochemical analysis of USP7 protein expression in TNBC using a patient tissue microarray with 110 TNBC tumors found that TNBC tumors had significantly higher levels of USP7 protein than normal breast tissue (Fig. 1D, E). Elevated USP7 protein levels were observed across all TNBC stages, reaching statistical significance compared to normal breast tissue in more advanced and invasive stages 2 and 3 (Fig. 1F).

We also analyzed breast cancer cell sensitivity to USP7 KO using the large-scale functional genomic screens available on the Cancer DepMap^21^ after subclassifying cells into known pathologic subtypes (ER+/Her2−, Her2+, and TNBC; Fig. 1G). This classification scheme is highly predictive of sensitivity to cell cycle inhibitors targeting CDK4/6, with TNBC cells being universally resistant^5^. TNBC cell lines are sensitive to USP7 KO, despite their recurrent loss of *TP53* (Fig. 1F). This supports the notion that USP7 could contribute to tumorigenesis through mechanisms that are independent of p53.

### USP7 promotes TNBC clonogenicity

**Fig. 2:**
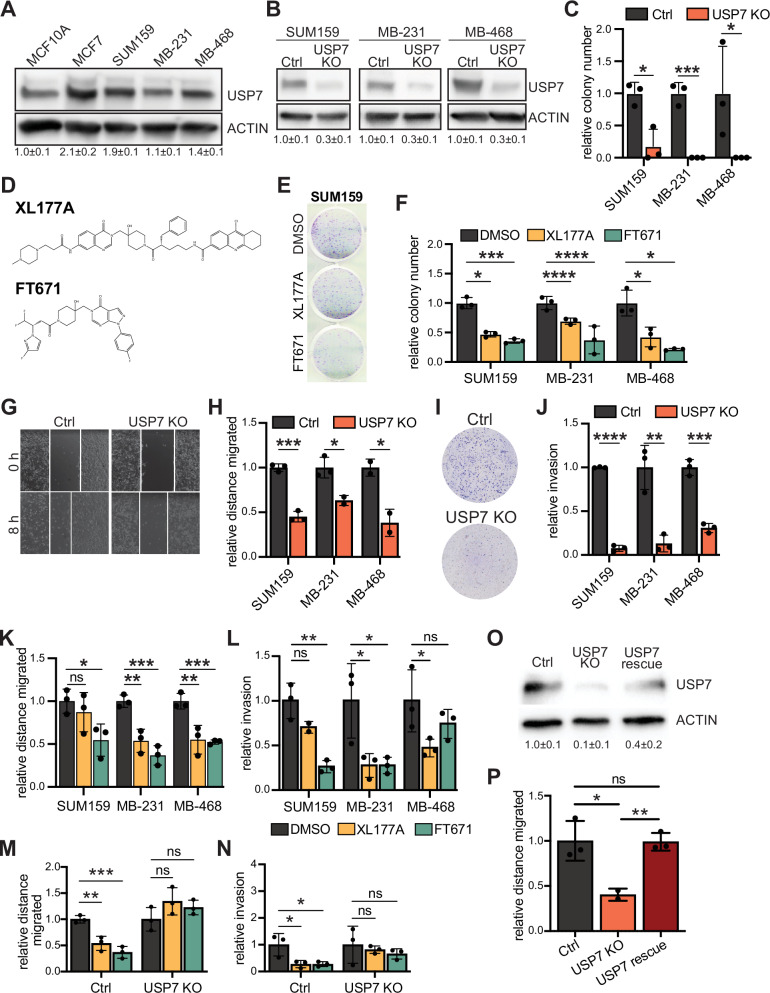
USP7 promotes TNBC clonogenicity, cell migration, and invasion. Representative Western blots (WB) showing USP7 protein levels in **A** malignant breast cancer cells, including TNBC, relative to MCF10A after β-actin normalization, and **B** CRISPR/Cas9-generated USP7 knockout (USP7 KO) TNBC clones compared to non-targeting controls (Ctrl). Quantification shown is mean ± SD (*n* = 3) independent WB. **C** Soft-agar colony counts for USP7 KO (red) vs Ctrl (gray). **D** Chemical structures of USP7 inhibitors used in this study. Representative images of soft-agar colonies in **E** SUM159 and **F** quantification of TNBC cells treated with XL177A (yellow) or FT671 (green). DMSO as control (green). Scratch-wound assay **G** images and **H** migration quantification for TNBC Ctrl and USP7 KO. Transwell invasion assays **I** images for SUM159 and **J** quantification comparing Ctrl and USP7 KO. Quantification of **K** migration and **L** invasion for TNBC cells treated with XL177A or FT671 (DMSO control). Quantification of **M** migration and **N** invasion for MDA-MB-231 Ctrl and USP7 KO treated with XL177A or FT671 (DMSO control). **O** WB for USP7 in MDA-MB-231 Ctrl (gray), USP7 KO clone (red), and USP7 rescue (maroon). USP7 abundance is shown relative to Ctrl. β-actin was used as a loading control. Quantification shown is mean ± SD (*n* = 3) independent WB. **P** Migration quantification for MDA-MB-231 Ctrl, USP7 KO, and USP7 rescue. All data normalized to control; bars represent mean ± SD (*n* = 3), dots represent independent replicates. Significance: ns, *p < 0.05, **p < 0.01, ***p < 0.001, ****p < 0.0001 by unpaired two-tailed *t*-test.

To investigate how elevated USP7 in TNBC increases malignancy, we selected three TNBC cell lines – SUM159, MDA-MB-231, and MDA-MB-468 – all of which were confirmed to express varying degrees of USP7 compared to the immortalized normal breast cell line MCF10A. We included the non-TNBC breast cancer cell line, MCF7, as a positive control (Fig. 2A)^11^. Using CRISPR/Cas9, we generated syngeneic *USP7* knockout (KO) clones and non-targeting control clones for each of the three TNBC cell lines (Fig. 2B and Supplementary Fig. 2). Proliferation analyses revealed a moderate but consistent increase in doubling time in USP7 KO clones compared to the control in the three TNBC lines (Supplementary Fig. 2). Of significance, USP7 KO conferred a significantly reduced ability to form colonies in clonogenic assays, with an average reduction of 94.0 ± 10.3% in the number of colonies formed compared to the control across the three cell lines (Fig. 2C). MDA-MB-231 and MDA-MB-468 cells lacking USP7 were almost completely unable to form colonies.

Highly selective USP7 small-molecule inhibitors have been extensively developed with nanomolar potency. For in vitro USP7 inhibition analyses, we used XL177A, an irreversible inhibitor with an IC50 of 0.34 nM^11^, and FT671, a noncovalent inhibitor with an IC50 of 52 nM^22^ (Fig. 2D). Both inhibitors have been screened against a panel of other DUBs, and both showed high specificity for USP7 inhibition^11,22^. USP7 pharmacological inhibition had minimal effects on cell viability and cell proliferation in vitro (Supplementary Fig. 3). However, similar to USP7 KO, treatment with XL177A or FT671 led to a significant reduction of 47.1 ± 14.4% and 68.5 ± 8.7%, respectively, in colony numbers across the three cell lines compared to DMSO (Fig. 2E, F).

These findings suggest that while USP7 has only a modest impact on cell proliferation in TNBC cell lines, it plays a critical role in sustaining clonogenic capacity, essential for long-term tumor growth and survival.

### USP7 promotes TNBC cell migration and invasion

Metastasis is the primary cause of breast cancer-related mortality and recurrence. USP7 plays a critical role in stabilizing proteins involved in metastasis^23,24^. A recent study also reported that USP7 promotes breast cancer migration and invasion, including in TNBC, through stabilization of the epigenetic reader ZMYND8^25^. In line with these observations, USP7 KO resulted in a significant decrease in cell migration in scratch-wound healing assays, with an average reduction of 47.2 ± 19.5% across the three TNBC cell lines (Fig. 2G, H). Invasion assays using Matrigel-coated inserts also revealed a significant decrease in invasiveness of 82.8 ± 12.1% in USP7 KO cells compared to control across all three TNBC cell lines (Fig. 2I, J).

Pharmacological inhibition of USP7 with FT671 similarly led to reduced cell migration in all three cell lines, while XL177A slowed migration in two out of three (Fig. 2K). USP7 inhibition also reduced the number of invaded cells compared to DMSO-treated controls in MDA-MB-231 (Fig. 2L). Although the reduction in invasion with USP7 inhibitors was less pronounced for SUM159 and MDA-MB-468, we observed a trend toward decreased invasion with USP7 inhibition (Fig. 2L). Confirming the on-target effects of both inhibitors, USP7 KO cells treated with USP7 inhibitors resulted in minimal changes in cell migration and invasion compared to control (Fig. 2M, N and Supplementary Fig. 3).

### USP7 drives tumor growth and metastasis in TNBC

**Fig. 3:**
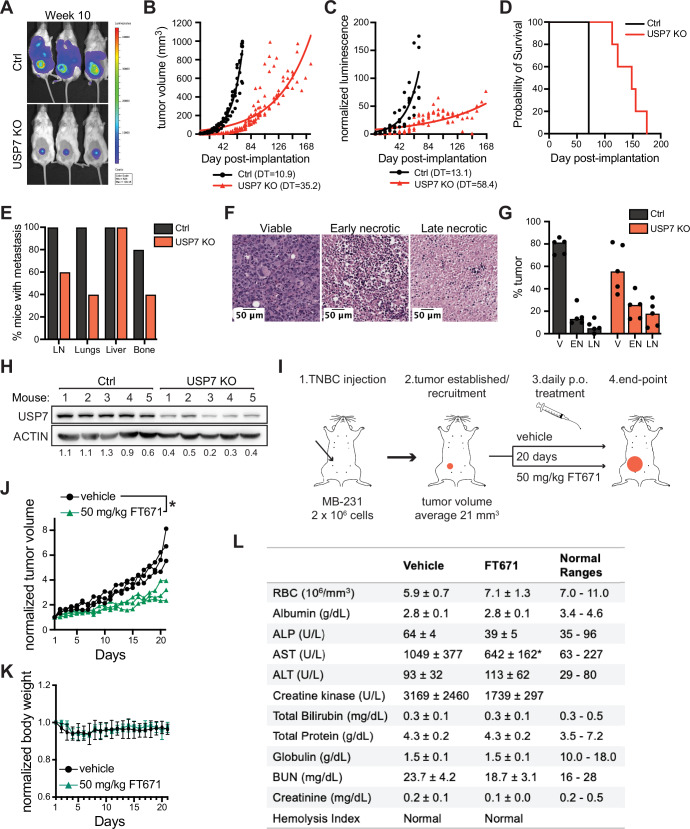
USP7 promotes TNBC tumor growth and metastasis. **A** Representative bioluminescent images of non-targeting vector control (Ctrl) and USP7 KO primary tumors in the mice at week 10. Overall intensity of bioluminescence is shown by area and color, with red having the highest photon count and blue the lowest. **B** Tumor growth curve comparing MDA-MB-231 Ctrl (*n* = 5; black) and USP7 KO (*n* = 5; red). Groups reached endpoint when the average tumor volume was 1000 mm^3^, measured by caliper. DT = doubling time in days. **C** Tumor growth curve comparing MDA-MB-231 Ctrl (black) and USP7 KO (red) measured by bioluminescence. DT = doubling time in days. **D** Survival curve comparing Ctrl and USP7 KO. Mice had reached endpoint based on the final average tumor volume of 1000 mm^3^. **E** Quantification of the percentage of mice bearing metastasis at endpoint. Black = Ctrl (*n* = 5); Red=USP7 KO (*n* = 5). LN = lymph nodes. **F** Representative histology sections of hematoxylin and eosin-stained tumors, distinguishing viable, early necrotic, and late necrotic tissue areas. **G** Percentage of viable, early necrotic, or late necrotic tumor areas comparing Ctrl to USP7 KO. **H** Western blot analysis for USP7 protein levels in Ctrl versus USP7 KO primary tumors. USP7 values normalized to control average after β-actin normalization. **I** Schematic workflow of mice bearing MDA-MB-231 tumors administered USP7 inhibitor, FT671, by oral gavage daily for 20 days. **J** Normalized tumor growth comparing vehicle (*n* = 3; black) and FT671 (*n* = 3; green) over 20 days of treatment. *p = 0.0038 by two-way RM ANOVA. **K** Daily average mouse body weight over 20 days of treatment for vehicle (black) and FT671-treated (green) mice. **L** Blood chemistry in the vehicle mice (*n* = 4) versus mice treated with 50 mg/kg FT671 (*n* = 3). Blood was collected after 20 days of daily treatment.

Building on the observed effects of USP7 on cell clonogenicity, migration, and invasion in TNBC, we next evaluated its role in promoting in vivo TNBC tumor growth and metastasis. NSG mice were orthotopically injected with MDA-MB-231, either control or USP7 KO, both expressing luciferase for bioluminescent monitoring. Tumors were tracked biweekly for 10 weeks. Tumor growth in the USP7 KO group was significantly slower than that of the control group. At the study endpoint, the USP7 KO group showed a significantly reduced tumor burden, based on bioluminescent imaging, and no detectable metastasis, whereas the control mice exhibited larger and highly metastatic tumors (Fig. 3A). Delayed tumor growth was also observed in MDA-MB-468-derived USP7 KO tumors (Supplementary Fig. 4A).

To account for differing tumor growth rates in assessing metastasis, a follow-up study set the endpoint to when tumor volumes reached 1000 mm^3^. USP7 KO tumors grew significantly slower, taking over 25 weeks to reach the endpoint compared to 10 weeks for control tumors (Fig. 3B, C). This delay in tumor growth translated to a significantly extended survival period for the USP7 KO group (Fig. 3D). Importantly, even when tumors reached comparable sizes, mice in the USP7 KO group exhibited significantly fewer metastatic lesions in lymph nodes, lungs, and bones compared to the control group (Fig. 3E). Interestingly, liver metastases were observed in all mice at comparable levels, suggesting that liver metastasis may be driven by USP7-independent factors such as chemokines and inflammatory signals^26^.

Transcriptomic analysis using bulk RNA-seq of MDA-MB-231 tumors revealed 1308 upregulated and 1053 downregulated genes in USP7 KO mice compared to controls (Supplementary Fig. 4B). Gene ontology (GO) analysis showed that the most enriched biological process for upregulated genes was negative chemotaxis, consistent with our observed reduced migration, invasion, and metastasis (Supplementary Fig. 4C). Downregulated genes, including TNF, were significantly associated with the negative regulation of extrinsic apoptotic signaling pathway in absence of ligand (Supplementary Fig. 4D). Histological analysis further supported these findings, with hematoxylin and eosin staining showing increased necrosis in USP7 KO tumors (Fig. 3F). These tumors displayed a significantly higher percentage of both early and late necrotic regions compared to controls (Fig. 3G). Western blot analysis confirmed reduced USP7 protein levels in the USP7 KO tumors (Fig. 3H). Altogether, these findings highlight USP7 as a critical driver of TNBC cell growth and metastasis.

### Pharmacological inhibition of USP7 slows TNBC tumor growth

Our findings demonstrated that genetic targeting of USP7 significantly reduces tumor growth. Building on the specificity of USP7 inhibitors observed in vitro, we investigated the therapeutic potential of FT671 in vivo. To evaluate the effects of USP7 inhibition, luciferase-expressing MDA-MB-231 cells were orthotopically injected into the mammary fat pads of NSG mice. Once tumors were established, mice were randomly assigned to receive daily oral (p.o.) treatment with either vehicle or 50 mg/kg FT671 (Fig. 3I). Tumor growth, measured daily by calipers, was significantly reduced in the FT671-treated group compared to the vehicle control over the 20-day treatment period (*p* = 0.0038) (Fig. 3J). Importantly, no differences in body weight were observed between the two groups, indicating that FT671 treatment was well tolerated (Fig. 3K). Toxicological analyses of the liver, heart, and muscle revealed no additional toxicity from FT671 treatment beyond the effects associated with DMSO, known to induce liver fibrosis (Fig. 3L). These results highlight the potential of USP7 inhibitors as a safe and effective therapeutic strategy for slowing TNBC tumor growth.

### Proteomics analysis uncovers TOPORS as a novel USP7 substrate

**Fig. 4:**
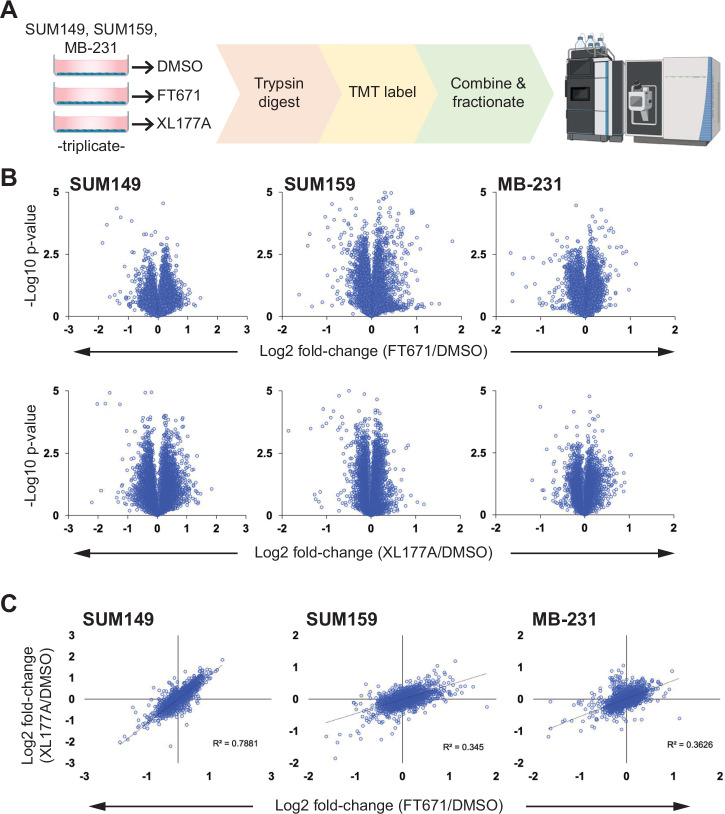
Deep, quantitative proteomics of USP7-inhibited TNBC cells. **A** Schematic describing the quantitative proteomics workflow for the USP7 inhibitor (USP7i) analysis. SUM149, SUM159, and MDA-MB-231 TNBC cell lines were grown and treated with DMSO, 1 μM FT671, or 1 μM XL177A for 8 h. Lysates are digested using trypsin, and LysC peptides are TMT labeled, mixed, and fractionated. Peptides are analyzed by mass spectrometry (MS), and protein abundances are determined. **B** Volcano plot showing the log2 fold-change (x-axis) and −log10 *p*-value (y-axis) of all proteins identified by MS when comparing samples from USP7i-treated (FT671 or XL1774A) cells to samples from DMSO-treated cells. Each dot represents an individual protein. **C** Plot showing the log2 fold-change ratio of all proteins identified by MS when comparing samples from FT671-treated to DMSO-treated cells (x-axis) vs the log2 fold-change ratio of all proteins identified by MS when comparing samples from XL177A-treated to DMSO-treated cells (y-axis).

To investigate the mechanisms underlying the poor outcomes associated with USP7 upregulation and the impact of USP7 inactivation on TNBC cells, we aimed to identify downstream ubiquitin signaling pathways leading to altered protein abundance that are affected by USP7. We employed TMT-based quantitative mass spectrometry to define the proteome-wide changes following USP7 inactivation. Three TNBC cell lines, MDA-MB-231, SUM159, and SUM149, were analyzed. All three were treated with either of the selective pharmacological USP7 inhibitors XL177A or FT671, or DMSO as a control, and profiled using multiplexed, quantitative proteomics using tandem mass tagging (Fig. 4A and Supplementary Data 1)^11,22^. To reveal proximal changes resulting from the direct regulation of USP7, which are most likely to be USP7 substrates, rather than secondary downstream alterations (e.g., changes in transcription factor expression), an acute, 8-h treatment duration was utilized for all cell lines and drug treatments. This approach allowed us to capture early proteomic alterations directly linked to USP7 activity.

We read deep into the proteomes of all three cell lines. After filtering for likely contaminants, we identified 7961 proteins in SUM149s, 8069 in SUM159, and 7845 proteins in MDA-MB-231. Collectively, we identified 8815 unique protein species between the three cell lines. Among those, 7845 proteins (~89%) were identified in more than one cell line, and 7102 (80%) were identified in all three cell lines. The high concordance between cell lines underscores their similarity based on their shared disease subtype and tissue of origin. Volcano plots for these 6 experimental conditions are shown in Fig. 4B. Importantly, comparison of FT671 to XL177A treatment revealed a high concordance, underscoring the previously reported, on-target activity of both inhibitors toward USP7, particularly notable given that these two drugs lack structural similarity (Fig. 4C).

**Fig. 5:**
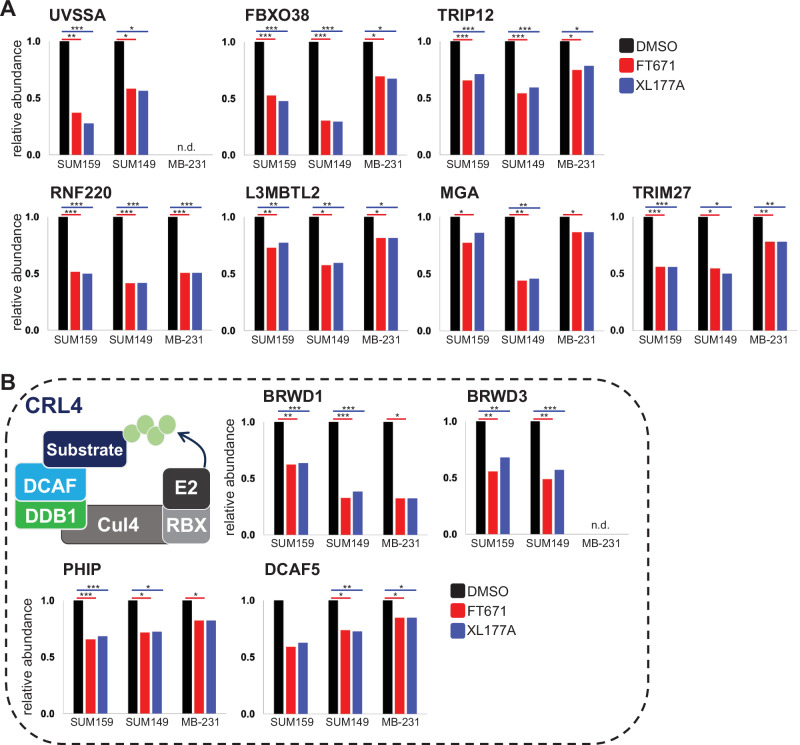
USP7 regulates diverse substrates, including several ubiquitin ligases. **A** Analysis of specific substrates from quantitative proteomics experiments performed in three cell lines and with two distinct USP7 inhibitors. The known substrates UVSSA, FBXO38, and TRIP12 were all significantly downregulated in response to USP7 inhibition in multiple cell lines, using both inhibitors. IN addition, several other proteins were downregulated across multiple cell lines, including RNF220, L3MBTL2, MGA, and TRIM27. **B** Several substrate receptors for the CRL4 ubiquitin ligase were similarly decreased by USP7 inhibition. A schematic of the modular CRL4 complex is shown. Interchangeable DDB1- and CUL4- associated factors (DCAFs) bridge substrates to the ubiquitin ligase complex by simultaneously binding DDB1 and ubiquitin targets. Among the substrate receptors controlled by acute USP7 inhibition are BRWD1, BRWD3, PHIP, and DCAF5. *p < 0.05, **p < 0.01, ***p < 0.001.

Proteomic datasets from SUM149, SUM159, and MDA-MB-231 cells were integrated, and proteins were ranked based on reproducibility across models, concordance of regulation across FT671 and XL177A, effect size, and statistical support. Proteins were retained if they met significance (*p* < 0.05) in at least one condition. Higher-ranking candidates were consistently detected across cell lines, showed uniform directionality of regulation, and exhibited stronger effect sizes with multiple significant comparisons, prioritizing robust and reproducible responses to USP7 inhibition. (Supplementary Data 1). Among the most significantly downregulated proteins were known USP7 substrates. This included UVSSA^27^, FBXO38^28^, TRIP12^29^, and TRIM27^30^ (Fig. 5A), although none have been previously reported in TNBC cell lines. We identified many other common hits that scored in multiple cell lines, many of which were E3 ligases. Among them was RNF220, a RING-finger ubiquitin ligase, which we validated by western blot (Fig. 5A and Supplementary Fig. 5A). L3MBTL2 is a chromatin-associated protein, and MGA is a dimerization partner with the oncogene MYC. Both were also consistently downregulated across cell lines treated with USP7 inhibitors (Fig. 5A). Surprisingly, we identified several substrate receptor proteins for the CUL4-based RING E3 ubiquitin ligase (CRL4). This modular family of E3 ligases uses CUL4 as a backbone and the adapter protein DDB1 to directly engage substrate receptors (see schematic in Fig. 5B). The CRL4 substrate receptors are often referred to as DCAFS, for DDB1- and CUL4-associated factors. Among those downregulated in multiple cell lines were BRWD1/DCAF19, BRWD3, PHIP/DCAF14, and DCAF5 (Fig. 5B). Collectively, the ability of USP7 to downregulate the abundance of many E3 ligases, including several DCAFs, following acute inactivation points to a broad role in coordinating proteostasis through the regulation of multiple E3 ligases. To validate proteins decreased by an orthogonal approach, we examined the abundance of proteins following USP7 depletion with two independent siRNAs. Western blotting for NUCKS1, TRIP12, and TOPORS, which were decreased across multiple cell lines based on proteomics experiments, showed a decrease in abundance after USP7 depletion with both siRNA, further supporting the proteomics data (Supplementary Fig. 5B).

**Fig. 6:**
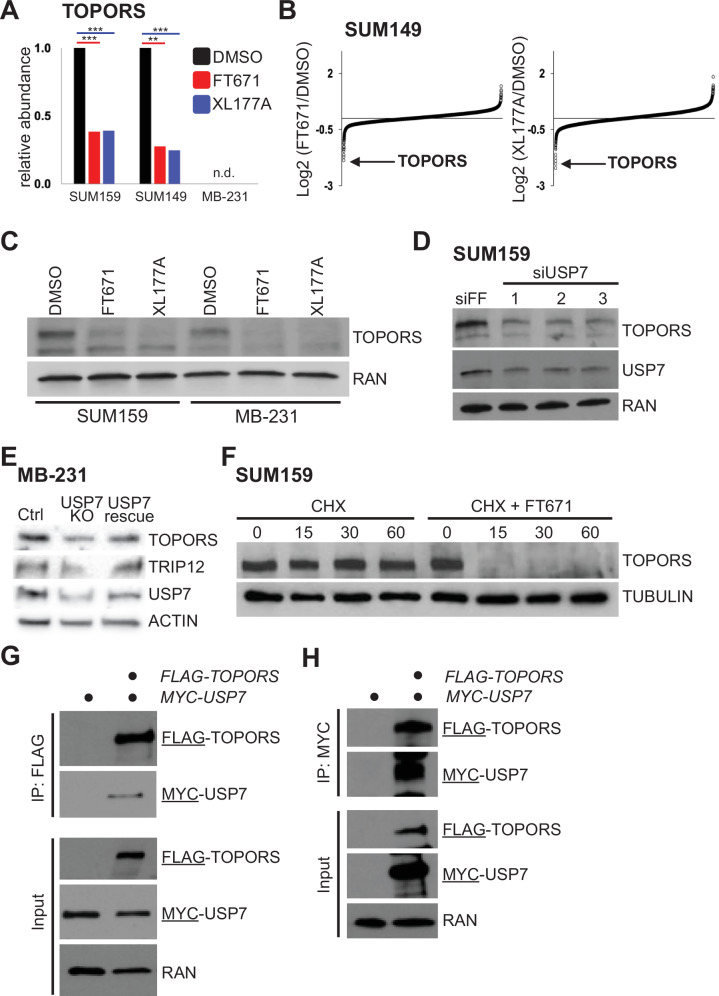
USP7 regulates TOPORS proteasomal degradation. **A** TOPORS was significantly downregulated by USP7 inhibition, quantitative proteomic analysis in response to FT671 and XL177A treatment in SUM149 and SUM159 cell lines. **p < 0.01, ***p < 0.001. **B** Plot showing the log2 ratio of all proteins identified by MS when comparing samples from FT671- (left) or XL177A-treated (right) to DMSO-treated SUM149 cells. Each dot along the x-axis represents an individual protein. **C**–**H** Representative Western blot analysis for **C** SUM159 and MDA-MB-231 cells treated with 1 μM of either FT671 or XL177A and analyzed for TOPORS; **D** SUM159 cells depleted of USP7 using siRNA and analyzed for the expression of TOPORS and TRIP12. **E** MDA-MB-231 Ctrl, USP7 KO, and USP7 rescue were analyzed for TOPORS, TRIP12, and USP7. β-actin was used as a loading control. **F** SUM159 cells treated with cycloheximide to block translation, or in the presence of FT671. The half-life of TOPORS was measured at the indicated time points, shown in minutes, following the inhibitor addition. **G**, **H** HEK293T cells transfected with indicated plasmids and analyzed by coIP for either FLAG-TOPORS (left) or MYC-USP7 (right).

Among the most downregulated proteins in multiple cell lines, TOPORS is a multifunctional protein implicated in cell growth, proliferation, DNA damage response, and ubiquitination. It functions as an E3 ubiquitin ligase and potentially an E3 SUMO ligase^31^. While its role remains poorly characterized, recent studies suggest it is involved in relieving DNA-protein crosslinks^32,33^. However, its function in TNBC has not been explored. Not previously reported as a direct USP7 substrate, TOPORS was significantly downregulated by both USP7 inhibitors in both SUM159 and SUM149 cells (Fig. 6A), although it was not detected in MDA-MB-231 cells. In SUM149 cells treated with FT671, TOPORS showed the most significant decrease among all 7961 proteins quantified and was the second most downregulated protein in SUM149 treated with XL177A (Fig. 6B). We validated TOPORS downregulation by western blot after pharmacologic USP7 inhibition in SUM159 and MDA-MB-231 cells (Fig. 6C). This decrease reflects an on-target effect of USP7 loss, as TOPORS reduction was similarly observed after USP7 depletion using multiple siRNAs and in USP7 KO cells, and could be rescued by re-expressing USP7 (Fig. 6D, E, Supplementary Fig. 5B). In addition, this decrease reflected destabilization of TOPORS, consistent with the notion that USP7 controls TOPORS ubiquitination and degradation since USP7 inhibition with FT671 shortened its half-life in a cycloheximide chase assay (Fig. 6E). We confirmed that this regulation is direct by assaying the interaction between USP7 and TOPORS using reciprocal co-immunoprecipitation in HEK293T cells (Fig. 6F, G), and importantly, by endogenous pulldown assays supporting a physiologically relevant interaction (Supplementary Fig. 5C).

### TOPORS associates with the BRCA1-A Complex

**Fig. 7:**
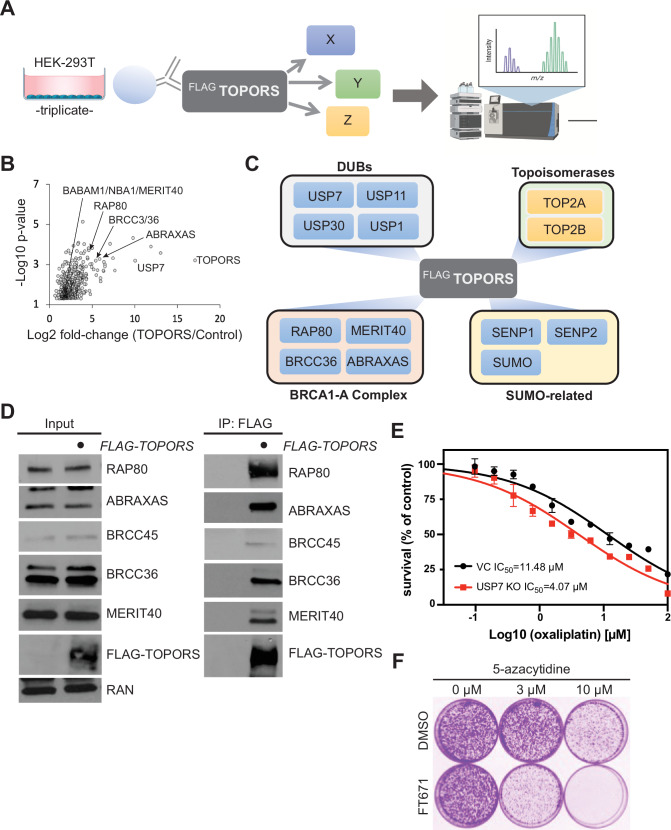
TOPORS interacts with the BRCA1-A complex. **A** TOPORS IP-MS schematic. FLAG-TOPORS was transfected into HEK293T cells in triplicate. FLAG-tagged TOPORS or an empty vector was immunoprecipitated, and samples were analyzed by LC-MS/MS. **B** Proteins enriched in TOPORS IPs compared to controls are shown. **C** TOPORS interactome showing selected interactor families identified by IP-MS. **D** HEK293T cells were transfected with FLAG-TOPORS or an empty vector control for 24 h, FLAG was immunoprecipitated, and eluates were analyzed by Western blot for the indicated proteins. **E** Dose-response curve for MDA-MB-231 USP7 KO (red) or vector control (black) cells treated with oxaliplatin continuously for 72 h. Each value represents the mean of 3 experiments ± SD; *p* < 0.0001. **F** Representative images for agar-free colonies stained with crystal violet comparing MDA-MB-231 treated with 1 μM FT671 and 0, 3, or 10 μM 5-azacytidine. DMSO was used as a vehicle control.

TOPORS has been linked to topoisomerase 1^34^ and has previously been shown to play a role in resolving DNA-protein crosslinks^32,33^. To gain deeper insight into its function, we carried out a protein interaction mass spectrometry screen (Fig. 7A and Supplementary Data 2). FLAG-TOPORS was expressed in triplicate samples of HEK293T cells, and following lysis and immunoprecipitation (IP), was analyzed by mass spectrometry. Control samples from cells expressing an empty vector were analyzed in parallel. USP7 was among the top-scoring interactors, further validating their interaction and the direct regulation of TOPORS by USP7 (Fig. 7B). Interestingly, several other DUBs were also identified, including USP11, USP30, and USP1. Several other interesting proteins were among the most enriched, including SUMO1 and two SUMO deconjugating enzymes, SENP1 and SENP2. Surprisingly, topoisomerases 2A and 2B were both enriched, although we did detect an enrichment for topoisomerase 1 (Fig. 7C).

Also, among the top interacting proteins were several components of the BRCA1-A complex (Fig. 7C). BRCA1 is a central mediator of DNA repair through homologous recombination, and significantly, is mutated in hereditary breast cancers that often develop into TNBC^35^. We identified RAP80, BABAM1/NBA1/MERIT40, BRCC3/BRCC36, and ABRAXAS1 as top interacting proteins. Using the FLAG-TOPORS expressing HEK293T cells, we validated the interaction between TOPORS and all the known components of the BRCA1-A complex using immunoprecipitation followed by Western blot analysis (Fig. 7D). We further validated this interaction by endogenous IP of TOPORS and blotting for ABRAXAS (Supplementary Fig. 5C). We also identified BRE/BRCC45, another component of the complex, only in our TOPORS pulldown, although we could not accurately quantify its enrichment. Remarkably, while we identified all the members of the BRCA1-A complex, we did not detect an interaction with BRCA1 itself, either by mass spectrometry or IP-western.

Collectively with published data, our results support a potential role for TOPORS in DNA damage repair. Interestingly, TOPORS depletion in SUM159 and MDA-MB-231 cells with siRNA had no significant impact on the levels of the BRCA1 complex proteins, suggesting that TOPORS might not control its degradation (Supplementary Fig. 6A). Nonetheless, our data, together with prior reports, link TOPORS to critical cancer pathways, with evidence suggesting that TOPORS loss sensitizes cells to DNA-damaging agents^36–38^. Because USP7 inhibitors destabilize TOPORS, we next evaluated whether USP7 inhibitors could enhance the response of genotoxic chemotherapy. Treatment with the USP7 inhibitor FT671 alone did not affect γH2A.X staining, but co-treatment with FT671 and camptothecin reduced γH2A.X levels (Supplementary Fig. 6B, C). In BRCA-proficient MDA-MB-231 cells, USP7 KO significantly increased sensitivity to oxaliplatin, reducing the IC50 from 11.48 µM in control cells to 4.07 µM in USP7 KO cells (*p* < 0.001; Fig. 7E). Colony assays further revealed increased susceptibility to 5-azacytidine when combined with FT671, indicating that USP7 inhibition enhances cellular response to multiple classes of genotoxic agents (Fig. 7F).

We also assessed the impact of PARP inhibition. Dose-response analysis showed no short-term effect of olaparib in USP7 KO cells (Supplementary Fig. 6D). However, in long-term colony formation assays, co-treatment with talazoparib and FT671 markedly reduced survival compared to either agent alone (Supplementary Fig. 6E).

Together, these findings suggest that USP7 inactivation sensitizes TNBC cells to DNA-damaging agents through TOPORS degradation and destabilization of the BRCA1-A complex.

## Discussion

USP7, a deubiquitinase that stabilizes numerous pro-tumorigenic proteins, has emerged as a critical oncogene in cancer biology. Our findings, alongside prior research, establish USP7 as a key regulator of tumor growth and metastasis in TNBC models through its regulation of protein stability and downstream signaling pathways^18,39,40^. Through genetic knockout and pharmacological inhibition, we demonstrated that targeting USP7 significantly impairs TNBC progression. Moreover, using deep quantitative proteomics, we identified several novel USP7-regulated targets, including TOPORS. These findings expand our understanding of USP7’s role in TNBC progression and highlight its potential as a therapeutic target. Targeting USP7 may exploit a context-specific vulnerability in TNBC by disrupting proteostasis and enhancing sensitivity to DNA-damaging therapies, offering a promising strategy for treating this aggressive breast cancer subtype.

Importantly, our data do not indicate that USP7 overexpression is unique to TNBC. Rather, we leverage TNBC as a biologically defined system with near-universal TP53 loss to uncover p53-independent functions of USP7. In our broad analysis of breast cancer patient data, we found USP7 is notably overexpressed at the mRNA level and frequently amplified at the chromosomal level, making it one of the most recurrently upregulated DUBs in the disease. Its upregulation is observed in nearly one-quarter of breast tumors, surpassing even the prevalence of CMYC alterations. USP7 overexpression occurs across all major breast cancer subtypes, including ER-positive, HER2- positive, and TNBC tumors, and is independent of *TP53* mutation status. Thus, in line with recent reports, USP7 likely contributes to tumorigenesis through p53-independent mechanisms^18,41,42^. This broad expression pattern, coupled with the association between high USP7 levels and poor patient outcomes, highlights its importance in breast cancer pathogenesis and progression.

Our in vivo studies revealed that USP7 KO tumors exhibit significantly reduced growth and metastasis to lymph nodes, lung, and bones, leading to extended survival. These findings emphasize USP7’s role in driving tumor aggressiveness. Furthermore, the increased necrosis observed in USP7 KO tumors indicates heightened tumor stress or impaired adaptation to the tumor microenvironment, warranting further investigation into the interaction between USP7 and the tumor microenvironment in metastatic progression. Pharmacological inhibition of USP7 using the selective inhibitor FT671 produced effects comparable to genetic ablation, with a modest but significant decrease in tumor growth without evident toxicity during treatment. These findings validate the clinical relevance of USP7 inhibitors as a therapeutic strategy in TNBC. Recently, additional strides have been made in the development of USP7 inhibitors, through various screening and chemical modification strategies^43–45^. Evaluation of these compounds’ ability to impede tumor growth, impact cancer phenotypes, and reshape proteomes in disease-specific models, while considering potential toxicities, is a critical next step in evaluating the therapeutic potential of USP7 in TNBC.

Mechanistically, our multi-omics approach revealed a broad regulatory role for USP7 in TNBC biology. Proteomic analysis identified USP7 as a master regulator of proteostasis, controlling the stability of multiple E3 ligases, including TRIP12^29^, TRIM27^30^, and TOPORS. Among these, TOPORS emerged as a novel USP7 substrate with critical roles in cell proliferation, DNA damage response, and relieving DNA-protein crosslinks^32–34,36,38^. While TOPORS’s function in TNBC has been unexplored, our findings indicate that USP7 directly stabilizes TOPORS, providing a mechanistic link between USP7 activity and pathways involved in genome integrity. TOPORS depletion did not alter the abundance of BRCA1-A complex proteins, suggesting that TOPORS, a dual ubiquitin and SUMO E3 ligase, does not regulate their stability under basal conditions^46,47^. However, both our data and prior studies implicate TOPORS in maintaining genome stability, and its loss has been associated with increased sensitivity to DNA-damaging agents^32^^,^^48^^,^^49^. Consistent with this, USP7 inhibition or genetic loss sensitized TNBC cells to multiple genotoxic chemotherapies, including oxaliplatin and 5-azacytidine. Although USP7 KO did not enhance acute sensitivity to PARP inhibition, long-term clonogenic assays revealed a significant decrease in survival when talazoparib was combined with FT671, indicating reduced proliferative capacity under sustained replication stress. These findings position TOPORS as a critical effector of USP7-mediated genome maintenance. They further suggest that therapeutic strategies combining USP7 inhibitors with replication-stalling or hypomethylating agents may enhance treatment efficacy in TNBC by exploiting vulnerabilities in DNA damage tolerance pathways.

Collectively, these findings position USP7 as a central regulator of TNBC biology, coordinating proteostasis, ubiquitination, and DNA damage repair. The identification of TOPORS as a USP7 substrate broadens the scope of USP7’s functional network and reveals an additional layer of regulation within the BRCA1-A-associated DNA repair machinery. These insights provide a mechanistic framework for the observed synergy between USP7 inhibition and genotoxic therapies, highlighting the potential of combining USP7 inhibitors with DNA-damaging or replication-stalling agents. Moreover, our results suggest that protein-based markers, such as BRCA1-A complex members, TOPORS, or USP7, could help guide therapeutic selection and stratify patients likely to benefit from these targeted combinations in TNBC.

Further studies are necessary to fully elucidate the spectrum of USP7-regulated pathways and to explore its role across different TNBC subtypes. Understanding the molecular contexts and biomarkers that predict USP7 inhibitor efficacy will be crucial for translating these findings into clinical applications, ultimately improving outcomes for patients with TNBC. Future studies should aim to elucidate the full spectrum of USP7-regulated pathways and explore its role in other TNBC subtypes. Understanding the molecular context in which USP7 inhibition is most effective will be critical for translating these findings into clinical applications.

## Methods

### Analysis of USP7 expression and amplification in the TCGA breast cancer cohort

We analyzed USP7 mRNA expression and copy number alterations using the Breast Invasive Carcinoma dataset from The Cancer Genome Atlas (TCGA, Firehose Legacy) accessed via cBioPortal. This cohort includes 1108 primary breast tumors with matched genomic and transcriptomic data. mRNA expression levels were derived from RNA sequencing data (RNA-Seq V2 RSEM) and are reported as z-scores normalized to tumors classified as diploid for USP7. A z-score threshold of ≥2.0 was used to define overexpression. Copy number alterations were assessed using GISTIC 2.0–processed data available through cBioPortal. Frequencies of USP7 amplification and mRNA overexpression were calculated across the cohort and compared to other deubiquitinases and oncogenes.

### Selection of TNBC cases from METABRIC and survival data

Survival annotated data from 2509 patients with breast cancer was obtained from the METABRIC study using cBioPortal on May 17, 2023^19,20^. We identified 199 patients with TNBC with associated mRNA microarray data. Data were stratified into quartiles (Q1-Q4, with Q1 representing the lowest expression) based on USP7 expression z-scores, and overall survival analysis was performed using Kaplan–Meier estimates and the Cox proportional hazards model. Overall survival information was provided in the data_clinical_patient.txt file for all 199 patients' IDs under OS_STATUS and VITAL_STATUS. We analyzed 10-year survival (120 months) with “Died of disease” cases flagged as events, while “Living” and “Died of Other Causes” cases were censored observations. Kaplan–Meier curves were plotted using the quartile stratification for USP7 expression group using the R package survival, and statistical significance was assessed using the log-rank test and defined as *p* < 0.05 to determine if there was a statistical difference between the highest and lowest quartile. A multivariable Cox proportional hazards model was used to determine if USP7 expression was independently associated with survival, controlling for age, stage, and intrinsic molecular subtype using the R package coxph.

### Immunohistochemistry

Tissue microarray (TMA) slides containing 110 TNBC cases were obtained from US Biomax (BRE1201; TissueArray.com). Slides were baked for 2 h at 60 °C, de-paraffinized with xylene, washed with ethanol, and hydrated. Slides were sub-boiled using 1× sodium citrate for 10 min and cooled. Sections were incubated with 3% hydrogen peroxide for 15 min, washed, and blocked using 5% goat serum in TBS-0.25% Tween (TBS-T) for 1 h at room temperature. USP7 (4833S; Cell Signaling Technology) primary antibody dilution (1:100 2% goat serum in TBS-T) was incubated overnight at 4 °C. The next day, slides were washed three times with TBS-T and incubated with 1:200 dilution of goat anti-rabbit biotinylated in 2% goat serum in TBS-T for 30 min at room temperature. Slides were washed three times with TBS-T and incubated for 30 min at room temperature with avidin/biotin-based peroxidase system (PK-6100; Vector Laboratories). After slides were washed three times with TBS-T, 3,3-diaminobenzidine (DAB) was added to stain slides for 2 min, followed by 10 s of hematoxylin counterstain. Slides were mounted using Gelvatol and scanned using Ventana DP200. All histological analyses were conducted using QuPath^50^.

### Cell culture

TNBC cell lines MDA-MB-231, MDA-MB-468, and SUM159, as well as the HER2-negative breast cancer cell line MCF7 and the immortalized non-malignant breast cell line MCF10A, were acquired from the American Type Culture Collection (ATCC). TNBC cell lines and MCF7 cells were cultured in Dulbecco’s modified Eagle’s medium (DMEM), high glucose (Gibco), and 10% fetal bovine serum (FBS; Gibco) with penicillin/streptomycin (P/S; Gibco). MCF10A cells were cultured in DMEM high glucose + 10% FBS with P/S and 20 μg/ml epidermal growth factor (EGF; Sigma-Aldrich). HEK293T cells were purchased from ATCC and cultured in high-glucose DMEM supplemented with 10% FBS and P/S. All cell lines were maintained in a humidified incubator at 37 °C with 5% CO_2_ and regularly screened for mycoplasma using a mycoplasma detection kit (13100-01, SouthernBiotech). All cell lines were routinely confirmed by STR profiling at the Arizona Genetics Core.

### Western blotting

Cell pellets or tumor tissues were lysed for 30 min on ice for protein extraction using RIPA buffer (50 mM Tris-HCl (pH 8), 150 mM NaCl, 1% NP-40, 0.5% sodium deoxycholate, 0.1% SDS, 1 mM EDTA) with protease inhibitor (100× Halt Protease Inhibitor Cocktail; Thermo Fisher). Lysates were centrifuged at 14,000 rpm for 30 min at 4 °C. The total protein concentration in the supernatant was quantified using a BCA protein assay (Pierce BCA Protein Assay Kit). The same concentration of total protein for lysates was loaded and resolved on a 4–15% gradient SDS-PAGE gel (Bio-Rad). Proteins were transferred onto a PVDF membrane (EMD Millipore) by semi-dry transfer. The membrane was incubated for 1 h at room temperature with 3% non-fat dry milk in TBS-T to block non-specific binding. Primary antibody was diluted in 0.5% non-fat dry milk in TBS-T at 1:1000 USP7 (4833S; Cell Signaling Technology) and 1:4000 β-actin (A2228; Sigma) and incubated overnight at 4 °C on a shaker. Following incubation, the membrane was washed twice with TBS-T and incubated with either goat anti-rabbit (PI-1000-1; Vector Laboratories) or horse anti-mouse (PI-2000-1; Vector Laboratories) HRP-conjugated secondary antibody diluted at 1:5000 in 0.5% non-fat dry milk in TBS-T and incubated for 30 min with shaking at room temperature. Membrane was washed twice with TBS-0.25% Tween, and bands were visualized using a chemiluminescent substrate (34580; Thermo Scientific) and imaged using Syngene G:BOX. The bands were quantified using ImageJ software.

Alternatively, cells were harvested in 1× PBS by gently scraping. The cells were pelleted by centrifuging at 3500 rpm for 5 min. The cell pellets were lysed in NETN (20 mM Tris pH 8.0, 100 mM NaCl, 0.5 mM EDTA, 0.5% NP-40) lysis buffer freshly supplemented with protease (1 mg/mL aprotinin, 1 mg/mL leupeptin, 1 mg/mL pepstatin) and phosphatase (25 mM Na3VO4, and 25 mM ABESF) inhibitors for 15 min on ice. The lysate was clarified by centrifuging at 15,000 rpm for 15 min. The total protein in the clarified cell lysate was measured using the Bradford assay (Bio-Rad). 25 μg of protein was separated using a 4–20% gradient gel (Bio-Rad) and proteins electroblotted to a nitrocellulose membrane at 100 V for 1 h. The blot was blocked for an hour, rinsed, and probed with appropriate antibodies overnight at 4°C. The blots were then rinsed in TBS-T three times, followed by incubation with an HRP-conjugated secondary antibody against the respective host for an hour at room temperature. The blots were washed with TBS-T three times, and the blot was incubated in ECL for 2 min. The chemiluminescence was captured using Amersham Hyperfilms and developed. The primary antibodies used were: 1:1000 TOPORS (A302-179A; Bethyl), 1:1000 USP7 (sc-30164; Santa Cruz), 1:1000 Rap80 (14466S; Cell Signaling Technology), 1:500 Abraxas (A302-180A; Bethyl), 1:1000 Merit40 (12711; Cell Signaling Technology), 1:1000 BRCC36 (18215; Cell Signaling Technology), BRCC45/BRE (12457; Cell Signaling Technology). 1:1000 Tubulin (sc-32293; Santa Cruz) or 1:1000 Ran (sc-271376; Santa Cruz) was used as loading control.

### Immunoprecipitation

HEK293T cells were transfected with 2 μg of plasmid encoding either Flag-TOPORS or Myc-USP7 or both using PolyJet (SignaGen). The transfection was performed overnight, followed by a media change, and the cells were incubated for another 24 h. The total length of transfection was 48 h. The cells were harvested on ice by scraping in 1× PBS. The harvested cells were pelleted and lysed in NETN lysis buffer. A protein quantification using the Bradford assay (Bio-Rad) was performed. To immunoprecipitate TOPORS or USP7, Flag or Myc beads were used, respectively. The beads (Sigma) were prepared by washing with PBS-T twice and then with cold NETN buffer. Equal amounts of protein from all groups were used for immunoprecipitation. The protein lysate and beads were nutated at 4 °C for an hour. The beads were collected by centrifugation and washed with cold lysis buffer three times. After the final wash, the bound protein was eluted using 2× Laemelli buffer by boiling at 100 °C for 5 min. The sample was then separated using SDS-PAGE.

### siRNA transfection

siRNA against TOPORS (siTOPORS sequence 1: GGUCUCGUAGCAGUGAUCAUGGUAA; siTOPORS sequence 2: UUACCAUGAUCACUGCUACGAGACC; Invitrogen) or USP7 (siUSP7 #1: CUAAGGACCCUGCAAAUUA; siUSP7 #2: GUGGUUACGUUAUCAAAUA; siUSP7 #3: UGACGUGUCUCUUGAUAAA; siUSP7 #4: GAAGGUACUUUAAGAGAUC; Sigma) was transfected using Lipofectamine RNAiMAX (Invitrogen) using the manufacturer’s protocol. Briefly, the cells were plated overnight before the transfection at a lower density. The siRNA and RNAiMAX were diluted to appropriate concentrations in OptiMEM (Invitrogen) in two separate tubes. The diluted RNAiMAX was added to the siRNA tube and incubated at room temperature for 15 min before adding to the cells. Before making the transfection mix, the culture media in the cells were changed to basal DMEM. The cells with the transfection mix were allowed to grow overnight. The next day, the media with the transfection mix was replaced with complete DMEM culture media, and cells were allowed to grow for another 24 h. An immunoblot was performed to confirm the depletion of either TOPORS or USP7.

### Lentivirus production and transduction

*USP7* gRNA (gRNA1 sequence: AGATGTATGATCCCAAAACG and gRNA2 sequence: GTGTACATGATGCCAACCGA) was cloned into the doxycycline-inducible CRISPR/Cas9 plasmid TLCV2 (87360; Addgene). pLenti PGK V5-LUC Neo (21471; Addgene) was used for bioluminescence imaging. All lentiviruses were produced by calcium phosphate transfection using HEK293T cells and the lentiviral TLCV2 transfer vector, lentiviral envelope plasmid pCMV-VSV-G (8454; Addgene), and lentiviral packaging plasmid pCMV-dr8.2-dvpr (8455; Addgene). Supernatants containing lentiviral particles were collected 24 and 48 h post-transfection and centrifuged at 1600 × *g* for 10 min at 4 °C to remove cell debris. Supernatants were filtered through 0.45 μm PES filter (ge-0504; Nalgene) and concentrated by ultracentrifugation at 23,000 rpm for 1.5 h at 4 °C. Lentiviral pellets were resuspended in cold PBS and stored in aliquots at −80 °C. Following lentiviral titering, transduction of target cells was achieved by exposing cells to viral particles in serum-free conditions for 6 h using a 0.2 MOI. Selection was carried out using either puromycin or G418, depending on the vector.

### CRISPR KO single cloning

The CRISPR KO pool of clones was generated by inducing SUM159, MDA-MB-231, and MDA-MB-468 TLCV2 *USP7* gRNA cells with 1 μg/ml doxycycline. 24- and 48-h post-induction, cells were checked for GFP expression, plated at a low density of 100–200 cells per 100 mm dish, and allowed to expand as single colonies for 3 weeks. The cell medium was removed from the dish, and 2% agarose with 2× PBS mixed in equal parts was poured onto the dish to solidify. Trimmed p200 pipette tips were used to puncture and draw up agarose and were vigorously pipetted up and down into a low volume of cell medium in each well, generating bubbles. The dispersed cells were allowed to adhere, recover, and expand as single clones. After numerous rounds of cell proliferation, single clones were screened for KO status using Western blotting. Single clones with the lowest protein expression levels of USP7 were selected for in vitro and in vivo studies. KO clones were assessed for CRISPR genome editing using Tracking of Indels by Decomposition (TIDE).

### Cell proliferation, colony formation assay

Cells were seeded at 10–20% confluency in 96-well plates and imaged every 24 h using a BioTek BioSpa and Cytation5 cell imaging reader. Cell proliferation was assessed by cell count quantification using ImageJ software.

For the soft-agar colony formation assay, 3000 cells were mixed in 0.36% agarose with full-serum media, and the top layer was allowed to solidify above the bottom layer containing 0.75% agarose in full media. Single cells were given five weeks to form colonies. Colonies embedded in agarose were fixed and stained with 0.1% crystal violet in 10% ethanol at room temperature for 15 min. Agarose was de-stained in multiple rounds of washing with Milli-Q water and imaged using a BZ-X700 Keyence microscope. The colonies were manually counted under a light microscope.

USP7 inhibitors, XL177A or FT671, were used at 1 μM (for SUM159 and MDA-MB-231) or 5 μM (for MDA-MB-468) and replenished every 24 h.

### Chemicals

USP7 inhibitors: XL177A (10 mM in DMSO) was kindly provided by Sara J. Buhrlage^11^. Stock solution was aliquoted and stored at −20 °C. FT671 was purchased from MedChemExpress (cat# HY-107985). Stock solution of 10 mM in DMSO was prepared, aliquoted, and stored at −80 °C.

### Scratch-wound healing assay

Cells were seeded as a monolayer in 24-well plates the day before the scratch to reach 70–80% confluency. 2 h prior scratch, cells were treated with 5 μg/ml mitomycin C (S8146; Sigma) to control for proliferation differences. Using a 200 μl pipette tip, the monolayer was scratched across the center of the well. Images were captured immediately (0 h) and 8 h after the wound at the same location. The distance between the boundaries of the wound at 0 and 8 h at 10 different locations was measured using ImageJ software. Assay was performed in triplicate, with each bar representing the mean ± standard deviation. For the migration assays with USP7 inhibitors, 1 μM (for SUM159 and MDA-MB-231) or 5 μM (for MDA-MB-468) XL177A or FT671 was added to the cells immediately after the scratch (0 h).

### Transwell invasion assay

Cells were treated with 5 μg/ml mitomycin C for 2 h at 37 °C. 8 μm pore PET membranes (0877121; Thermo Fisher) were coated with 25 μg of Matrigel per insert and were incubated for 1 h at 37 °C to solidify prior to adding a layer of cells resuspended in serum-free media. Full-serum medium containing 100 ng/ml FGF-2 was added to the wells beneath the inserts. Cells were allowed to invade the Matrigel overnight at 37 °C on the bottom side of the insert. The remaining cells in the inner chamber were swabbed, and cells on the bottom side of the insert were fixed with 70% ethanol and stained with 0.2% crystal violet. The inserts were de-stained with Milli-Q water and imaged using BioTek Cytation5. The images were stitched using the ImageJ software, and the stained cells were manually counted. Assay was performed in triplicate, with each bar representing the mean ± standard deviation. For the invasion assays with USP7 inhibitors, 1 μM (for SUM159 and MDA-MB-231) or 5 μM (for MDA-MB-468) XL177A or FT671 was added with the cells onto the Matrigel.

### Animal studies

NOD *scid* gamma (NSG) mice were acquired from Jackson Laboratory. 8-to-10-week-old NSG female mice were injected orthotopically under the mammary fat pad located below the fourth nipple with 50 μl of 2 × 10^6^ cells resuspended in sterile 1× PBS. Surgery was performed on anesthetized and shaved mice. Using tweezers to lift the skin around the nipple, the fat pad was visible under the light. A 27G tuberculin syringe with an attached needle was then used to enter the skin a few millimeters from the center of the nipple, and cells were slowly injected under the center. After the needle was slowly removed, the injection site was observed for signs of leakage. The mice were removed from anesthesia and allowed time to recover post-surgery. Tumor sizes were measured biweekly either by caliper measurement or bioluminescence imaging (IVIS Lumina). Primary tumors, lungs, and visible metastatic tumors were collected and used for analysis. To confirm the presence of metastasis, ex vivo imaging was conducted on the lymph nodes, lungs, liver, and bones.

For oral gavage, FT671 was dissolved in 10% DMSO/90% PEG400. Mice received a lead-in dose of 25 mg/kg of FT671 or vehicle when the tumors had reached an average volume of 168 mm^3^. Mice were then dosed daily for 20 days at 50 mg/kg of FT671 or vehicle. At the study endpoint, primary tumors and lungs were collected for analysis. Blood was collected by cardiac puncture; 10 μl was immediately mixed with 10 μl 100 mM EDTA and analyzed for complete blood count, and the remaining blood was left to clot for 30 min at room temperature. The blood sample was spun at 2000 × *g* for 10 min at 4 °C to collect the supernatant (serum). Serum was analyzed via Standard Tox Panel (62794; IDEXX BioAnalytics).

The University of California, Irvine Institutional Animal Care and Use Committee approved all animal procedures (AUP-23-058).

### RNA sequencing

Total RNA was isolated using RNA Microprep Kit (R1050; Zymo Research) or RNeasy Mini Kit (74104; Qiagen). 500 ng of total RNA was used to enrich for poly(A) mRNA to generate an RNA sequencing library using NEBNext Ultra II Directional RNA Library Kit for Illumina per manufacturer’s protocol (E7760L; New England Biolabs). Samples were purified with AMPure XP beads (A63881; Beckman Coulter) and eluted in 20 μl of 0.1× TE buffer. Samples were checked for library quality using Agilent BioAnalyzer 2100 and quantified using KAPA qPCR. A paired-end 100-bp sequencing run was conducted on Illumina NovaSeq X Plus with 50 million PE reads.

### Proteomics

SUM159, SUM149, and MDA-MB-231 cells were treated with either DMSO, 1 μM FT671, or 1 μM XL177A for 8 h prior to harvesting for global quantitative proteomics at the UNC Proteomics Core Facility. Briefly, treated cells were harvested and washed with ice-cold 1× PBS three times. Cells were kept on ice or chilled throughout. Next, cells were lysed using urea lysis buffer (8 M Urea, 75 mM NaCl, 50 mM Tris, pH 8.0, 1 mM EDTA, 1× protease inhibitor cocktail, and 1× phosphatase inhibitor cocktail) for 10 min on ice and were subsequently snap frozen in liquid nitrogen. Cell lysates were spun down to remove debris for 15 min at maximum speed in a refrigerated microcentrifuge. Protein concentration was determined by Bradford Assay. Lysates were stored at −80 °C prior to submitting for analysis to the Proteomics Core. All analyses were performed in triplicate.

TMT Proteomics Analysis. Sample Preparation: Protein lysates (300 µg) were reduced with 5 mM DTT for 45 min at 37 °C and alkylated with 15 mM iodoacetamide for 30 min in the dark at room temperature. Samples were digested with LysC (Wako, 1:50 w/w) for 2 h at 37°C, then diluted to 1 M urea and digested with trypsin (Promega, 1:50 w/w) overnight at 37 °C. The resulting peptide samples were acidified to 0.5% trifluoracetic acid, desalted using desalting spin columns (Thermo), and the eluates were dried via vacuum centrifugation. Peptide concentration was determined using a Fluorometric Peptide Assay (Pierce). Samples were split into three sets (*n* = 9 each) based on cell line, and labeled with TMT10plex reagents (Thermo Fisher). A pooled sample, consisting of ~2 µg of each sample, was used to fill the extra TMT channel. 40 µg of each sample was reconstituted with 50 mM HEPES pH 8.5, then individually labeled with 150 µg of TMT reagent for 1 h at room temperature. Prior to quenching, the labeling efficiency was evaluated by LC-MS/MS analysis of the pooled sample. After confirming >98% efficiency, samples were quenched with 50% hydroxylamine to a final concentration of 0.4%. Labeled peptide samples were combined 1:1, desalted using a Thermo desalting spin column, and dried via vacuum centrifugation. The two dried TMT-labeled samples were fractionated using high pH reversed-phase HPLC. Briefly, the samples were offline fractionated over a 90 min run, into 96 fractions by high pH reverse-phase HPLC (Agilent 1260) using an Agilent Zorbax 300 Extend-C18 column (3.5-µm, 4.6 × 250 mm) with mobile phase A containing 4.5 mM ammonium formate (pH 10) in 2% (vol/vol) LC-MS grade acetonitrile, and mobile phase B containing 4.5 mM ammonium formate (pH 10) in 90% (vol/vol) LC-MS grade acetonitrile. The 96 resulting fractions were then concatenated in a non-continuous manner into 24 fractions and dried down via vacuum centrifugation and stored at −80 °C until further analysis.

### LC/MS/MS Analysis

The samples, consisting of 2 sets of 24 fractions (48 fractions total), were analyzed by LC/MS/MS using an Ultimate 3000-Exploris480 (Thermo Scientific). Samples were injected onto an IonOpticks Aurora series 2 C18 column (75 μm id × 15 cm, 1.6 μm particle size), and the samples were separated over a 140 min method. The gradient for separation consisted of 5–45% mobile phase B at a 250 nl/min flow rate, where mobile phase A was 0.1% formic acid in water and mobile phase B consisted of 0.1% formic acid in 80% acetonitrile. The Exploris480 was operated in turboTMT10 mode with a cycle time of 3 s. Resolution for the precursor scan (m/z 375–1400) was set to 60,000 with an AGC target set to 100%; maximum injection time set to auto. Resolution of the MS2 scan was set to 30,000, and consisted of HCD normalized collision energy (NCE) 33; AGC target set to 300%; maximum injection time of 55 ms; isolation window of 0.7 Da.

### TMT proteomics data analysis

Raw data were processed using Proteome Discoverer (version 2.5) for peptide/protein identification and TMT10plex quantitation. Data were searched against a Uniprot Reviewed Human database (downloaded 01/2022, containing 20,360 sequences), appended with a common contaminants database, using the Sequest HT search engine. A maximum of two missed tryptic cleavages was allowed, and fixed modifications were set to TMTpro peptide N-terminus and Lys and carbamidomethyl Cys. Dynamic modifications were set to N-terminal protein acetylation and oxidation of Met. Precursor mass tolerance was set to 10 ppm, and fragment mass tolerance was set to 0.02 Da. MS2 coisolation interference threshold was set to 30%. The peptide false discovery rate was set to 1%. Reporter ion abundance was calculated based on intensity. Results were filtered to 5% protein FDR, a minimum of 2 peptides, and quantitation in minimum 50% of samples. Statistical analysis (Student’s *t*-test, unpaired) was conducted within Argonaut^51^. Proteins with an absolute log2 fold-change ≥0.5 and a *p*-value < 0.05 are considered significant (Supplementary Data 1). The mass spectrometry proteomics data have been deposited to the ProteomeXchange Consortium via the PRIDE partner repository^52^ with the dataset identifier PXD059902.

### Proteomic data integration and candidate prioritization

Proteomic datasets from three TNBC cell lines (SUM149, SUM159, and MDA-MB-231) were integrated into a single matrix using gene name as the common identifier. For each protein, all corresponding measurements across cell lines and treatment conditions (FT671 and XL177A) were retained, and missing values were left unassigned when a protein was not detected in a given dataset. Protein accession numbers and descriptions were preserved throughout the integration process. All log₂ fold-change and *p*-value columns were converted to numeric format, and non-numeric entries were treated as missing values. Proteins were retained for downstream analysis if they met statistical significance in at least one condition, defined as *p* < 0.05 in any comparison. No additional filtering based on fold-change thresholds was applied at this stage to avoid excluding moderate but potentially reproducible effects. For each protein, four summary metrics were computed to guide prioritization. Reproducibility across models was estimated as the number of non-missing log₂ fold-change measurements normalized to the number of inhibitor conditions, providing an approximation of the number of cell lines in which the protein was detected. Concordance of regulation was defined globally based on directionality, whereby proteins were considered concordant if all non-missing log₂ fold-change values shared the same sign. The effect size was calculated as the mean absolute log₂ fold-change across all available conditions. Statistical support was summarized using both the number of significant comparisons (*p* < 0.05) and the mean *p*-value across all non-missing conditions.

A composite prioritization score was calculated for each protein using a weighted linear model: score = (reproducibility × 3) + (concordance × 2) + (effect size × 1.5) + (number of significant comparisons × 1) − (mean *p*-value). Proteins were ranked in descending order of this score, prioritizing candidates that demonstrated consistent, concordant, and statistically supported regulation across multiple models and inhibitor conditions (Supplementary Data 1).

### Sample preparation for affinity purification mass spectrometry analysis (AP-MS)

Immunoprecipitated samples from FLAG-TOPORS or empty vector control pulldowns, performed in triplicate in HEK293T cells (one 10 cm dish per sample), were subjected to on-bead trypsin digestion, as previously described^53^. After the last wash buffer step, 50 µl of 50 mM ammonium bicarbonate (pH 8) containing 1 µg trypsin (Promega) was added to the beads overnight at 37 °C with shaking. The next day, 500 ng of trypsin was added, then incubated for an additional 3 h at 37 °C with shaking. Supernatants from pelleted beads were transferred, then beads were washed twice with 50 µl LC/MS grade water. These rinses were combined with the original supernatant, then acidified to 2% formic acid. Peptides were desalted with peptide desalting spin columns (Thermo) and dried via vacuum centrifugation. Peptide samples were stored at −80 °C until further analysis.

### LC/MS/MS analysis for TOPORS AP-MS

Each sample was analyzed by LC-MS/MS using an Easy nLC 1200 coupled to a QExactive HF (Thermo Scientific). Samples were injected onto an IonOpticks Aurora Elite TS C18 column (75 μm id × 15 cm, 1.7 μm particle size) and separated over a 120 min method. The gradient for separation consisted of a step gradient from 5 to 36 to 48% mobile phase B at a 250 nl/min flow rate, where mobile phase A was 0.1% formic acid in water and mobile phase B consisted of 0.1% formic acid in 80% ACN. The QExactive HF was operated in data-dependent mode, where the 15 most intense precursors were selected for subsequent HCD fragmentation. Resolution for the precursor scan (m/z 350–1700) was set to 60,000 with a target value of 3 × 106 ions, 100 ms inject time. MS/MS scans resolution was set to 15,000 with a target value of 1 × 105 ions, 75 ms inject time. The normalized collision energy was set to 27% for HCD, with an isolation window of 1.6 m/z. Peptide match was set to preferred, and precursors with unknown charge or a charge state of 1 and ≥ 8 were excluded.

### Data analysis for TOPORS AP-MS

Raw data were processed using the MaxQuant software suite (version 1.6.15.0) for peptide/protein identification and label-free quantitation^54^. Data were searched against a Uniprot Reviewed Human database (downloaded 01/2023, containing 20,404 sequences), appended with the HepaCAM sequences, using the integrated Andromeda search engine. A maximum of two missed tryptic cleavages was allowed. The variable modifications specified were: N-terminal acetylation and oxidation of Met. Label-free quantitation (LFQ) was enabled. Results were filtered to 1% FDR at the unique peptide level and grouped into proteins within MaxQuant. Match between runs was enabled. Data filtering and statistical analysis was performed in Perseus software (version 1.6.14.0) (Supplementary Data 2)^55^. The mass spectrometry proteomics data have been deposited to the ProteomeXchange Consortium via the PRIDE partner^52^ repository with the dataset identifier PXD059910.

### Statistical analysis

All graphs and statistical analyses were performed using the GraphPad Prism software (Prism 10 for macOS, GraphPad Software). The sample size (*n*), statistical tests, and *p-*values are noted in the figure legends. Results are presented as the mean ± SD. Statistical significance of differences between two groups in vitro was assessed by Student’s *t*-test, with *p*-values < 0.05 statistically significant. A two-way ANOVA was conducted for animal studies. Blind analysis was performed for the phenotypic measurements.

## Supplementary information

41523_2026_974_MOESM1_ESM

41523_2026_974_MOESM2_ESM

41523_2026_974_MOESM3_ESM

## Data Availability

The mass spectrometry proteomics data have been deposited to the ProteomeXchange Consortium via the PRIDE partner repository^52^ with the dataset identifiers PXD059902 and PXD059910. Other datasets generated and/or analyzed during the current study are available from the corresponding author on reasonable request.
