# Supplementary material for: USP7 inhibition perturbs proteostasis and tumorigenesis in triple-negative breast cancer
Source: NPJ Breast Cancer. 2026 May 23;12:107. doi: 10.1038/s41523-026-00974-5 (PMC13473141; doi:10.1038/s41523-026-00974-5)
Supplement: Supplementary file 1 [file 41523_2026_974_MOESM1_ESM.pdf]

Kim A. et al, Supplemental Figure 1

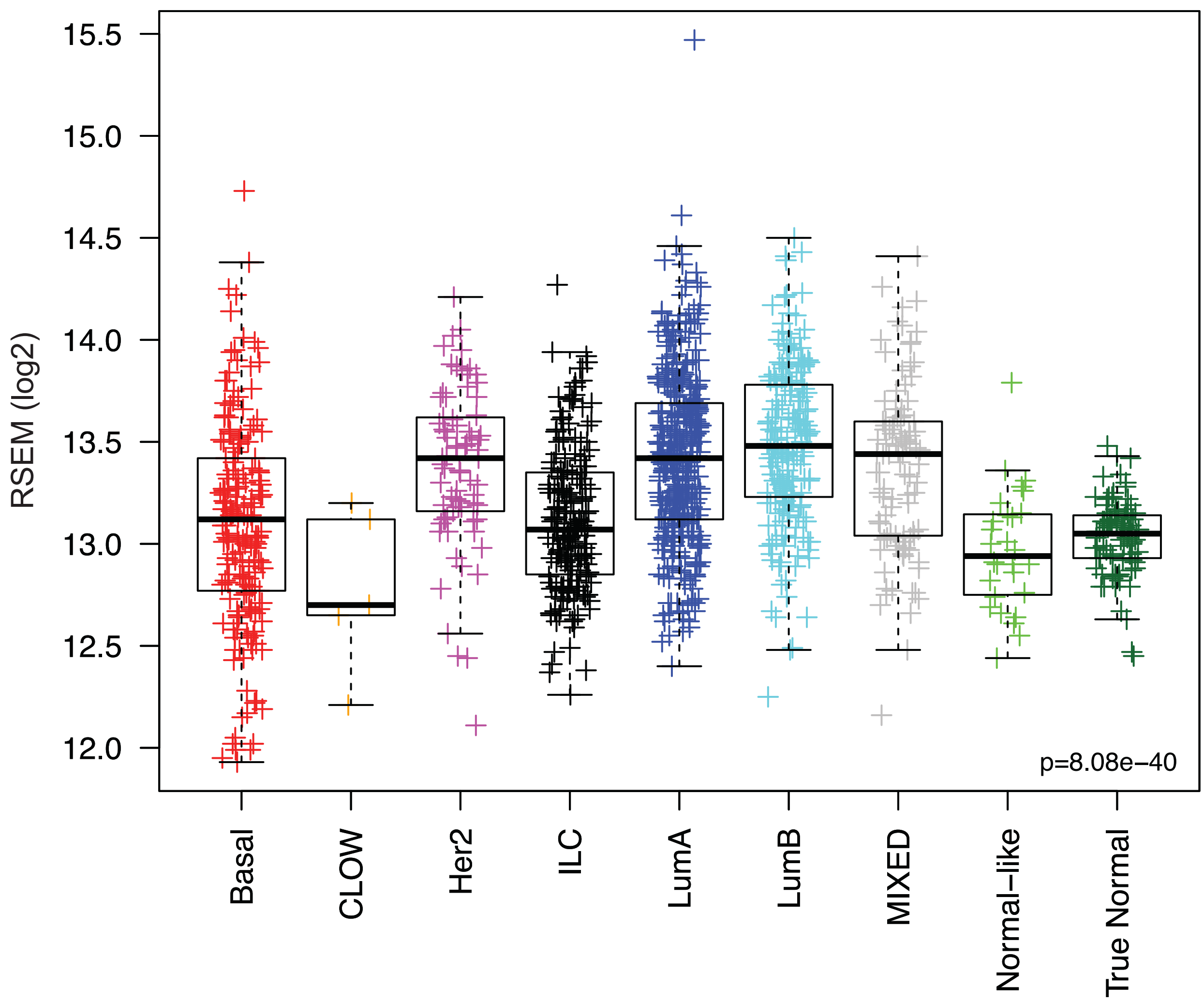

Supplemental Figure 1. Analysis of USP7 gene expression from the TCGA database comparing basal, Claudin-low (CLOW), human epidermal growth factor receptor negative (Her2), invasive lobular carcinoma (ILC), ER+ luminal A (LumA), ER+ luminal B (LumB), or a combination of more than one histologic type (MIXED) breast cancer subtypes to normal (true normal) and normal-like breast tissue.

Kim A. et al, Supplemental Figure 2

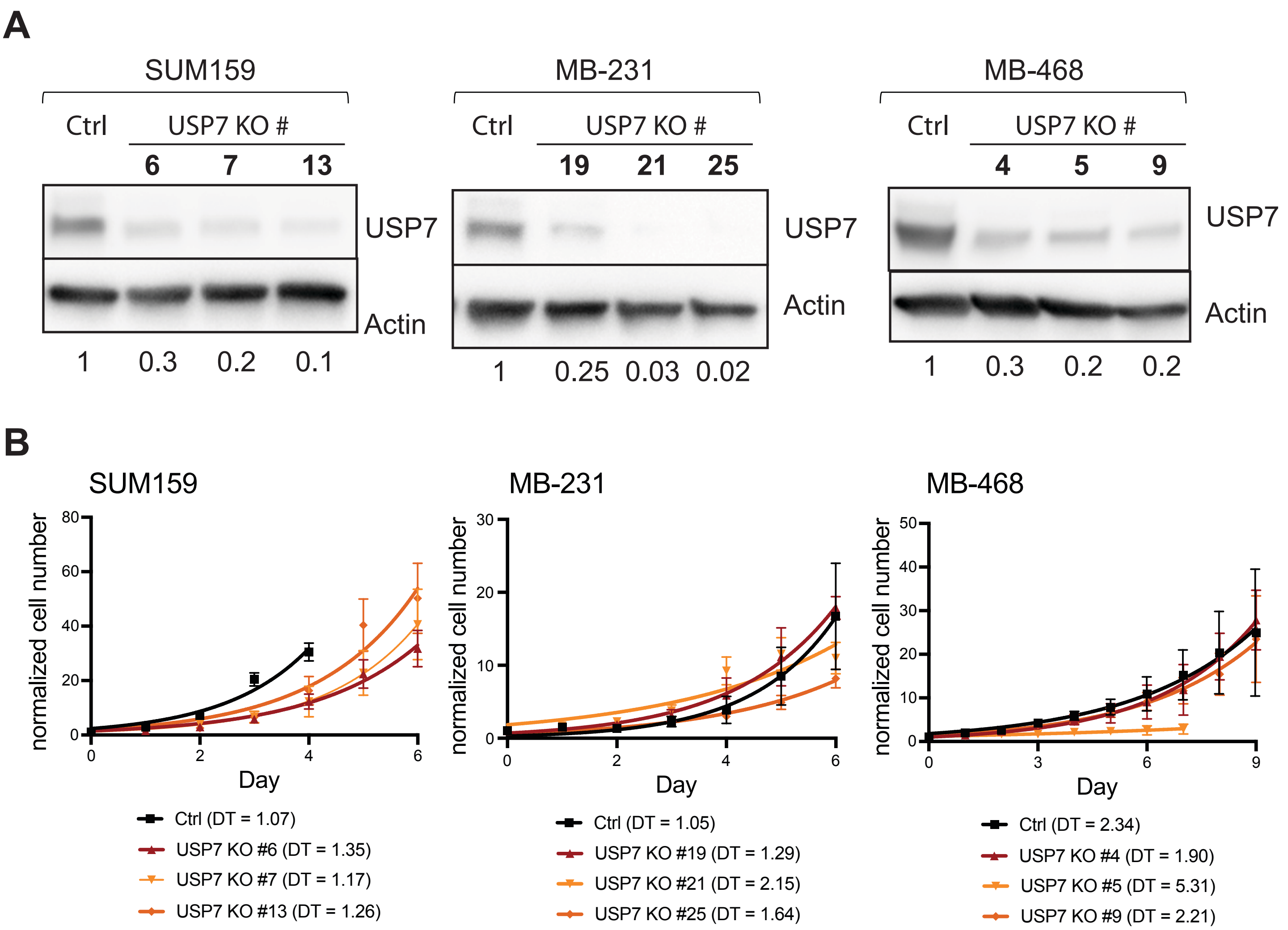

Supplemental Figure 2. (A) Western blot analysis of USP7 in numerous CRISPR/Cas9-mediated USP7 knockouts of TNBC cell lines (USP7 KO) in comparison to non-targeting vector control (Ctrl). β-actin was used as loading control. (B) Cell proliferation analysis for individual USP7 KO clones compared to Ctrl. Values were normalized to cell number at Day 0. Each data point is shown as mean ± SD, run in technical replicates (Ctrl n=3) and different clone numbers as biological replicates. DT = doubling time in days.

Kim A. et al, Supplemental Figure 3

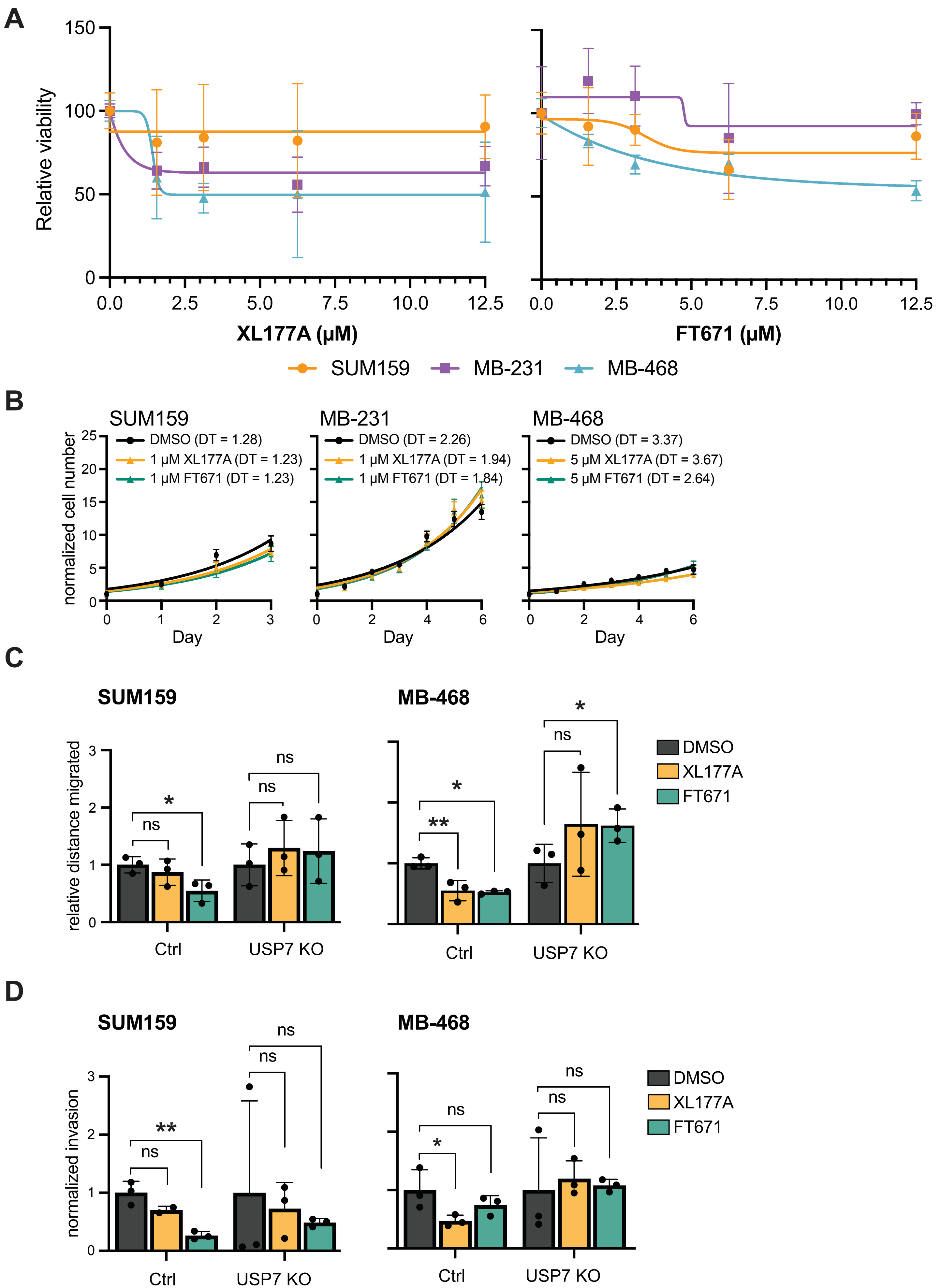

Supplemental Figure 3. (A) 72-hour viability curves showing range of concentrations for XL177A or FT671 in three TNBC cell lines. (B) Cell proliferation analysis for TNBC cells treated with XL177A or FT671. DMSO used as vehicle control. DT = doubling time in days. (C) Quantification of the distance cells migrated in scratch-wound assays normalized to DMSO for SUM159 or MDA-MB-468 Ctrl and USP7 KO treated with XL177A or FT671. Each data point is mean ± SD of biological replicates n=3. (D) Quantification of cell invasion across the Transwell membrane normalized to DMSO for SUM159 or MDA-MB-468 Ctrl and USP7 KO treated with XL177A or FT671. Each data point is mean ± SD of biological replicates n=3. For all bar graphs: ns=not significant, $*p < 0.05$, $**p < 0.01$ by unpaired two-tailed t test.

Kim A. et al, Supplemental Figure 4

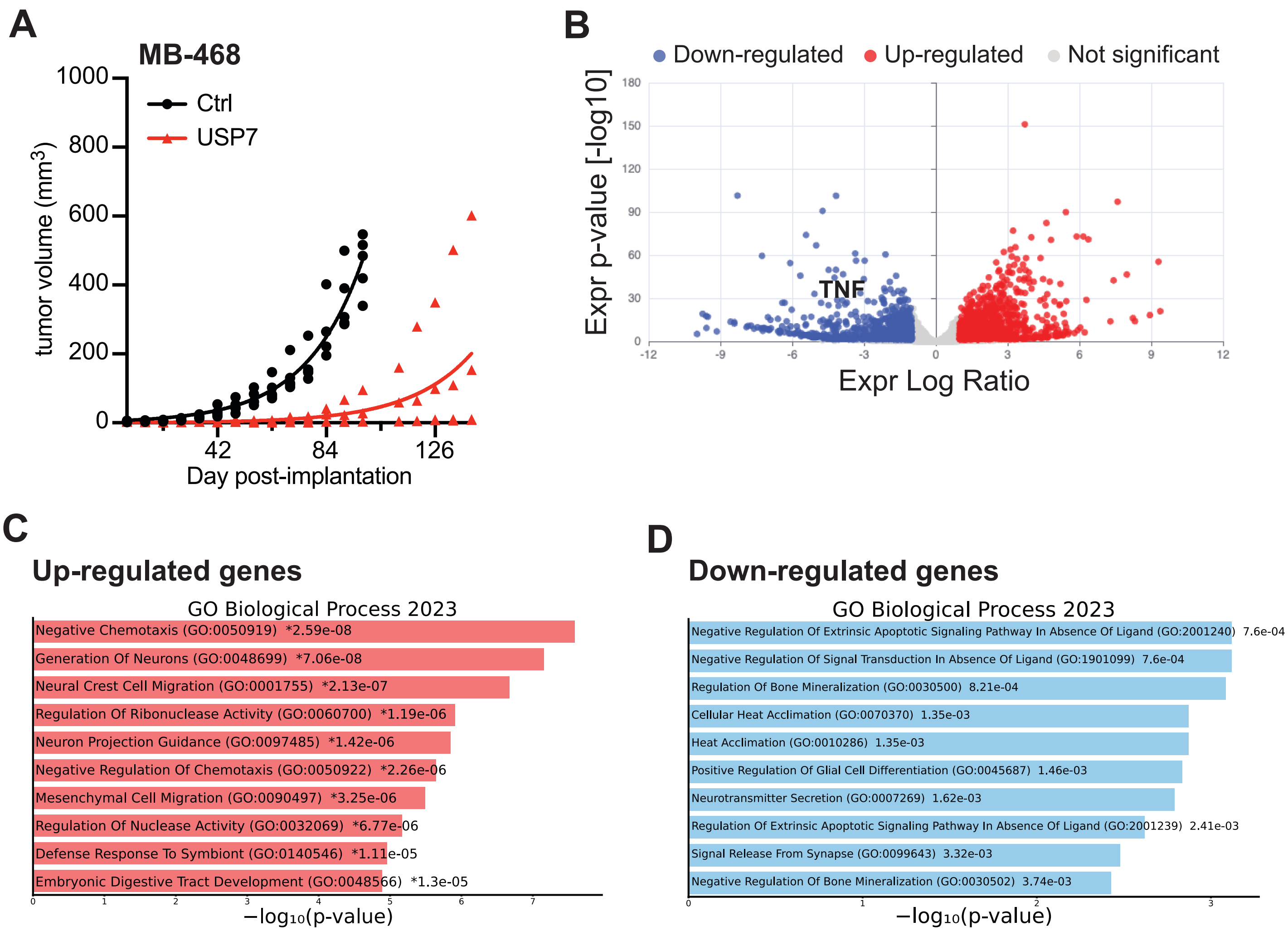

Supplemental Figure 4. (A) Tumor growth curve comparing MDA-MB-468 Ctrl (n=5; black) and USP7 KO (n=4; red). Groups reached endpoint when average tumor volume was 600 mm^3 measured by caliper or at 140 days. (B) Volcano plot depicting differentially expressed genes (DEG) from USP7 KO vs control tumors, where blue dots represent downregulated genes, red dots upregulated genes, and grey dots genes that did not meet significance based on expression log ratio <-1.0, >1.0 and expression p-value <0.05. (C-D) Gene ontology (GO) biological process analysis for (C) upregulated and (D) downregulated DEGs in USP7 KO vs control tumors identified through RNA-seq.

Kim A. et al, Supplemental Figure 5

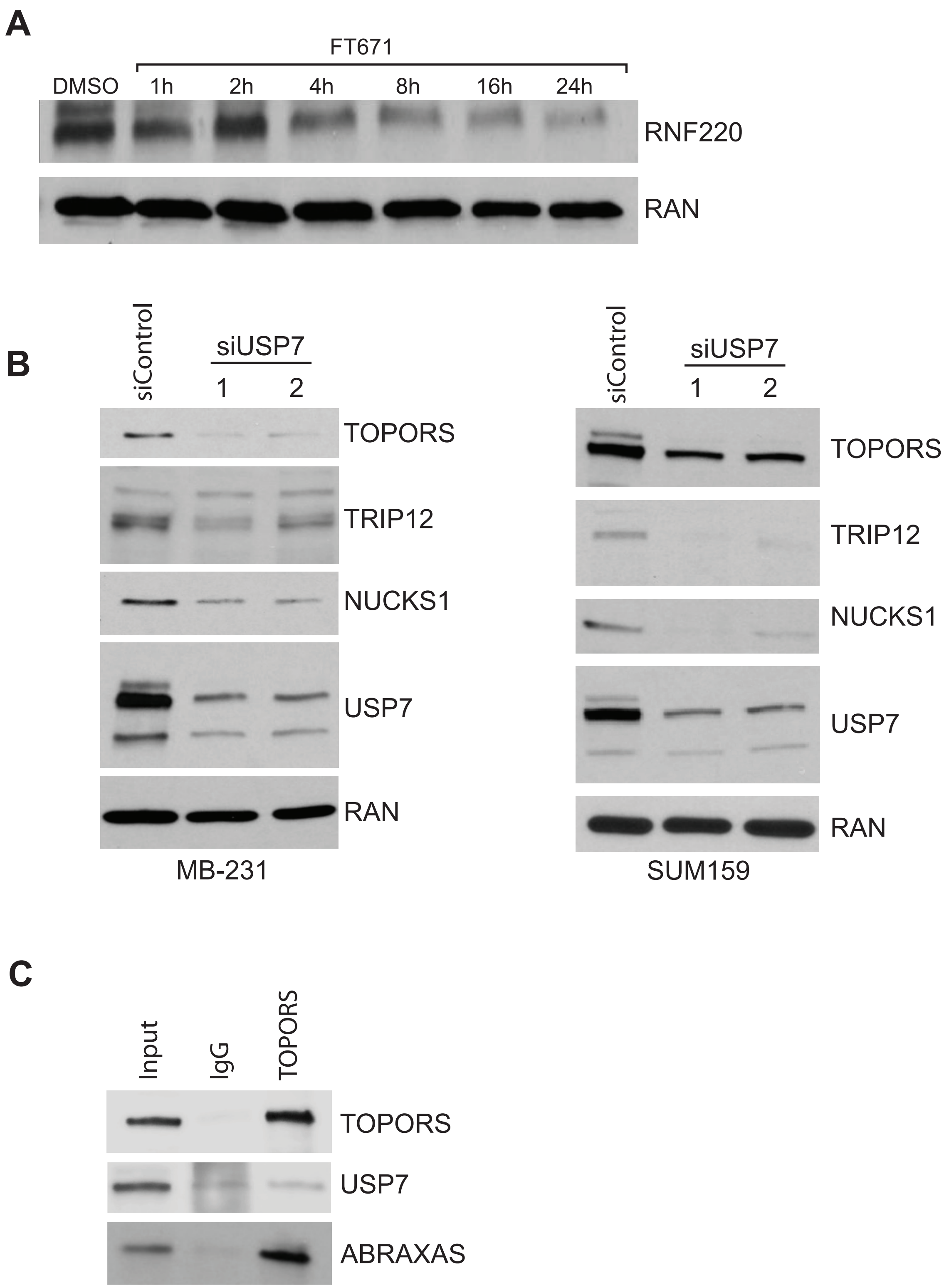

Supplemental Figure 5. Representative Western blot analysis for (A) RNF220 following a time-course treatment with FT671. RAN used as loading control. (B) Cells depleted of USP7 using siRNA and analyzed for the expression of TOPORS and TRIP12. (E) MDA-MB-231 Ctrl, USP7 KO, and USP7 rescue analyzed for TOPORS, TRIP12, NUCKS1, and USP7. β-actin used as loading control. (C) Immunoprecipitation analysis following TOPORS pulldown or IgG as control for TOPORS, USP7, and ABRAXAS using endogenous protein expression.

Kim A. et al, Supplemental Figure 6

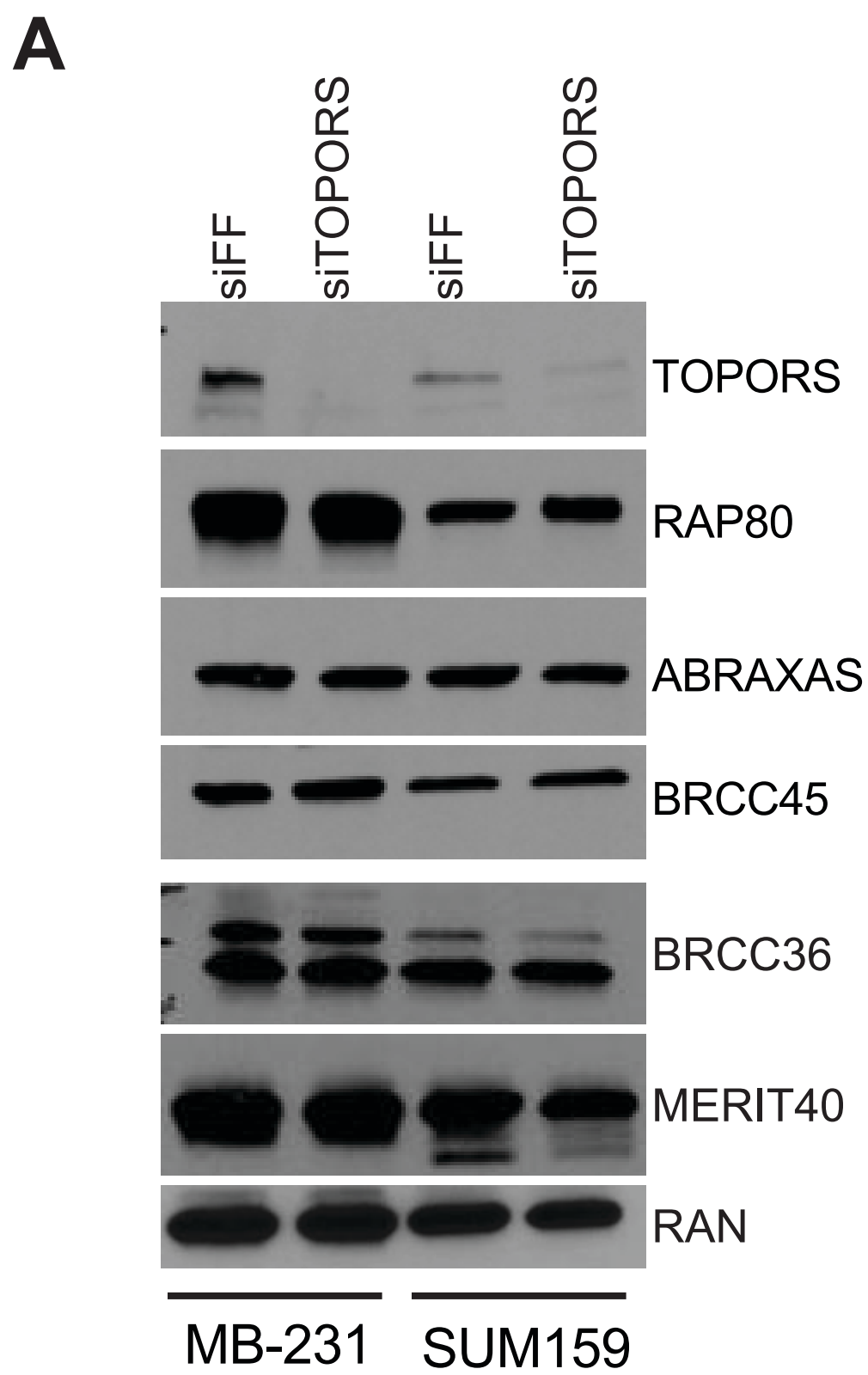

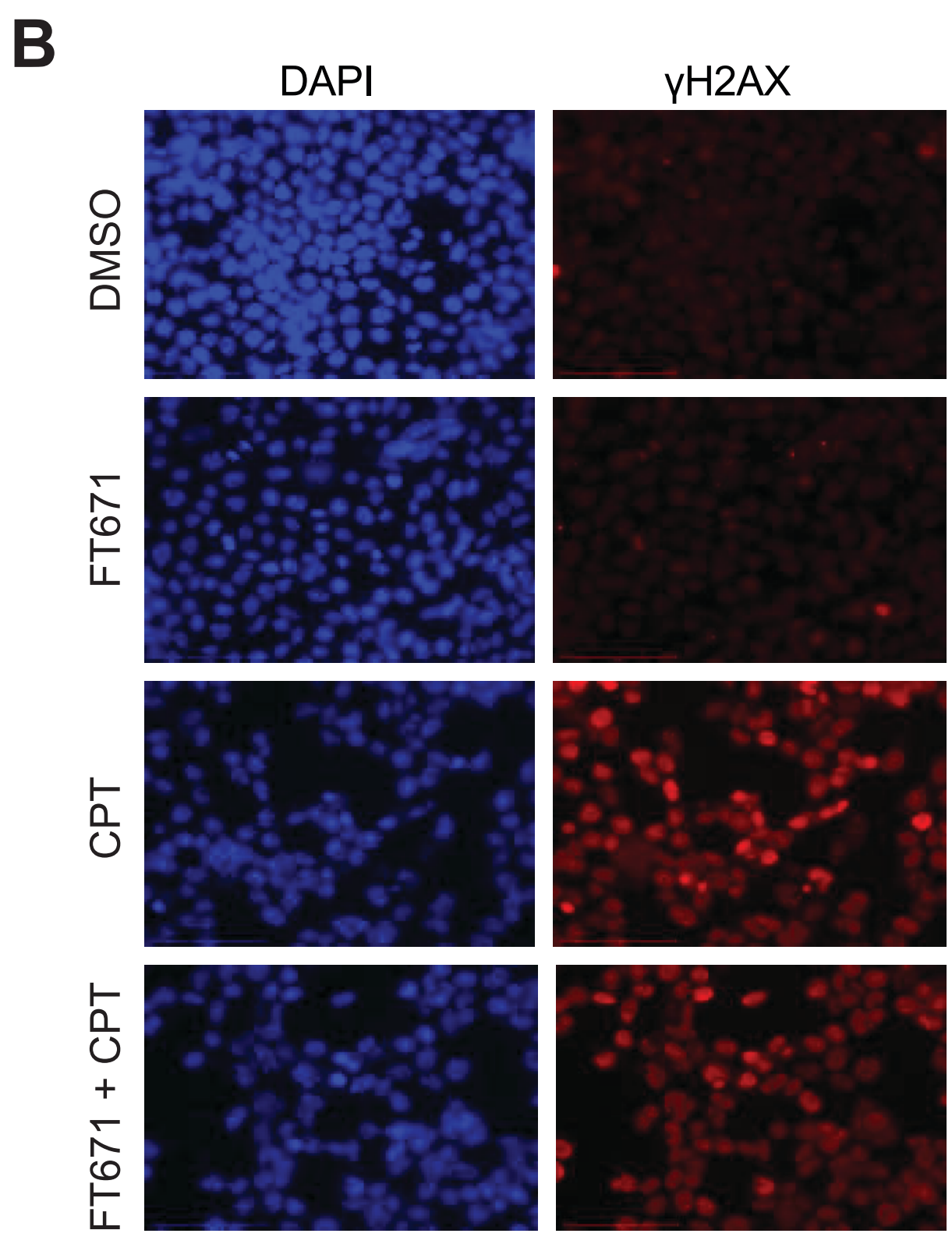

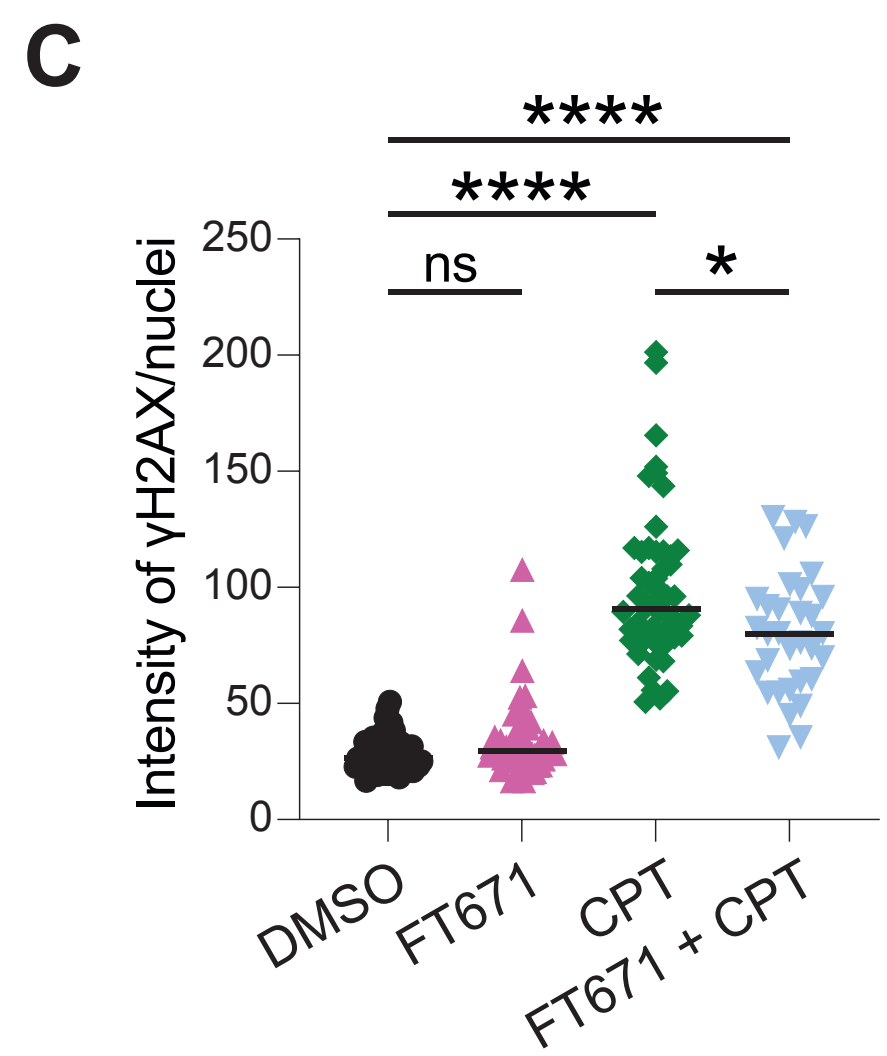

Supplemental Figure 6. (A) Western blot analysis of MDA-MB-231 and SUM159 TNBC cells treated with control (siFF) or TOPORS-targeted siRNA (siTOPORS). RAN used as loading control. (B) Representative immunofluorescence images of MDA-MB-231 cells treated with DMSO, 1 μM FT671, 1 μM camptothecin (CPT) or FT671 + CPT and stained using a γH2A.X antibody (red) and DAPI (blue). (C) Quantification of γH2A.X intensity per nuclei. Each dot represents a nucleus with the mean of 50 nuclei shown by the line. ns=not significant, *$p < 0.05$, ****$p < 0.001$ by unpaired two-tailed t test.

Supplemental Data 1. Tandem Mass Tag Mass Spectrometry Data for USP7 inhibitors in TNBC cell lines

Excel table summarizing Tandem Mass Tag (TMT) mass spectrometry data for triple-negative breast cancer (TNBC) cell lines SUM159, SUM149, and MDA-MB-231 treated with the USP7 inhibitors XL177A or FT671, or DMSO vehicle control. For each protein, the table reports $\log_2$-transformed TMT intensities, $\log_2$ fold changes for XL177A/DMSO and FT671/DMSO, and associated statistical significance values (p-values). Data enable comparative assessment of global proteomic alterations induced by distinct USP7 inhibitors across TNBC models.

Supplemental Data 2. Affinity Purification-Mass Spectrometry for TOPORS vs FLAG Control

Excel table containing quantitative affinity purification–mass spectrometry data for TOPORS immunoprecipitation relative to FLAG control, including enrichment metrics and statistical values used to define TOPORS-associated proteins.

Kim A. et al, Unprocessed immunoblots

Figure 2A

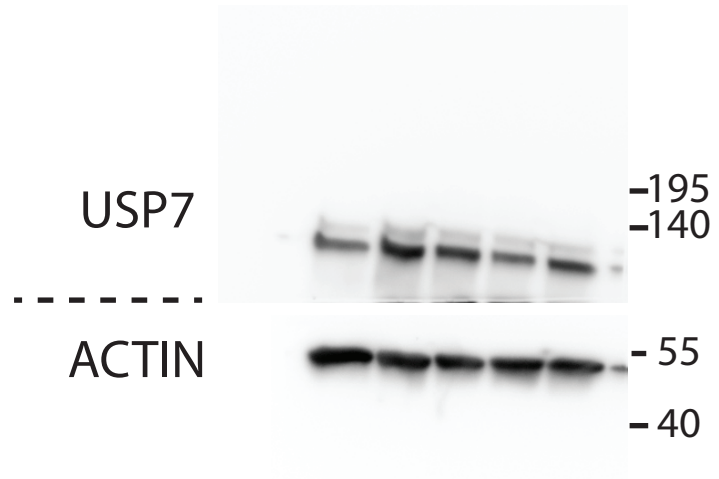

Figure 2B and Sup 2A

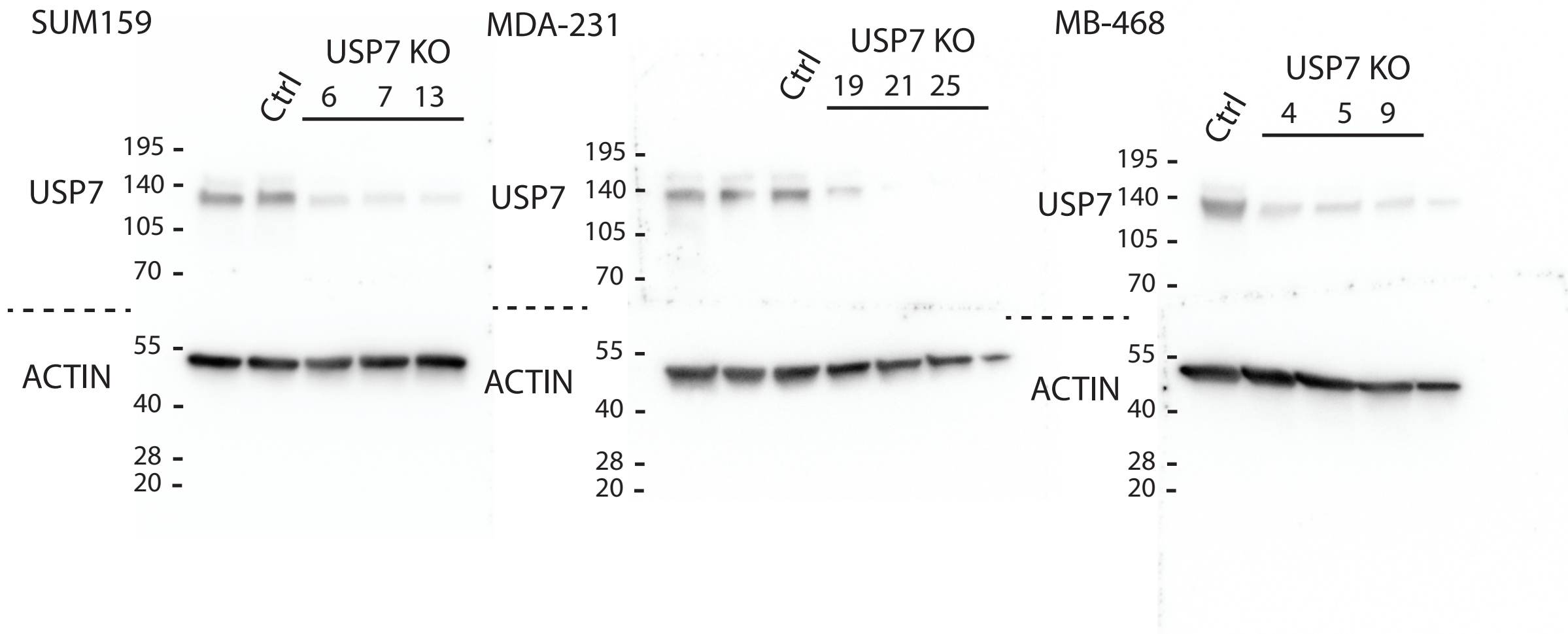

Figure 2O

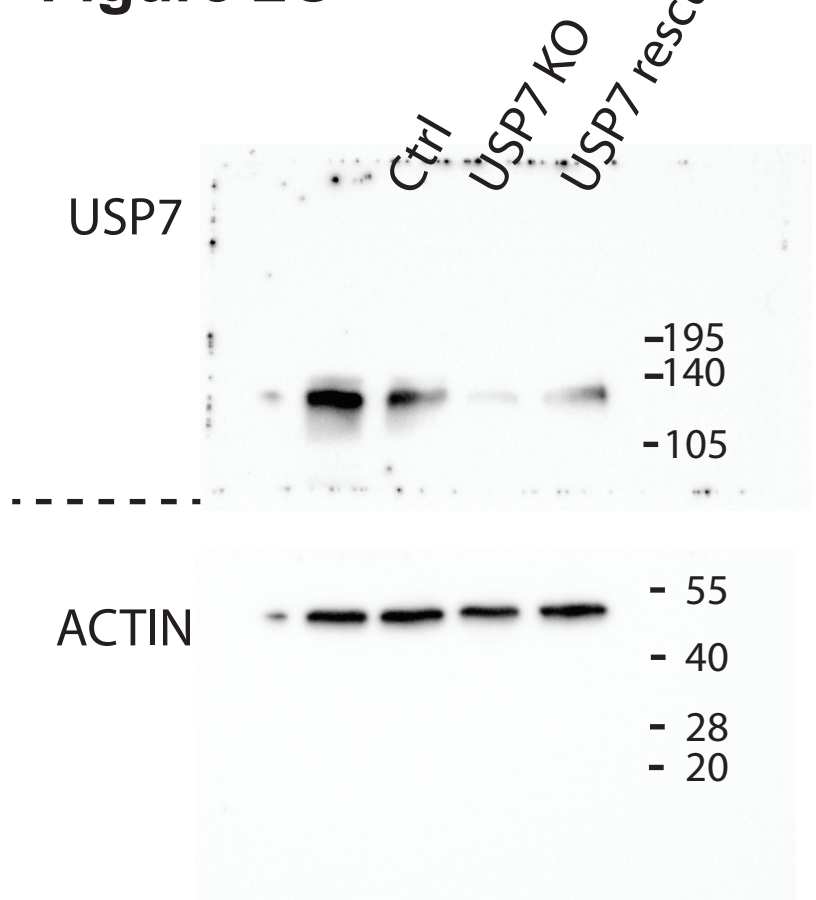

Figure 3H

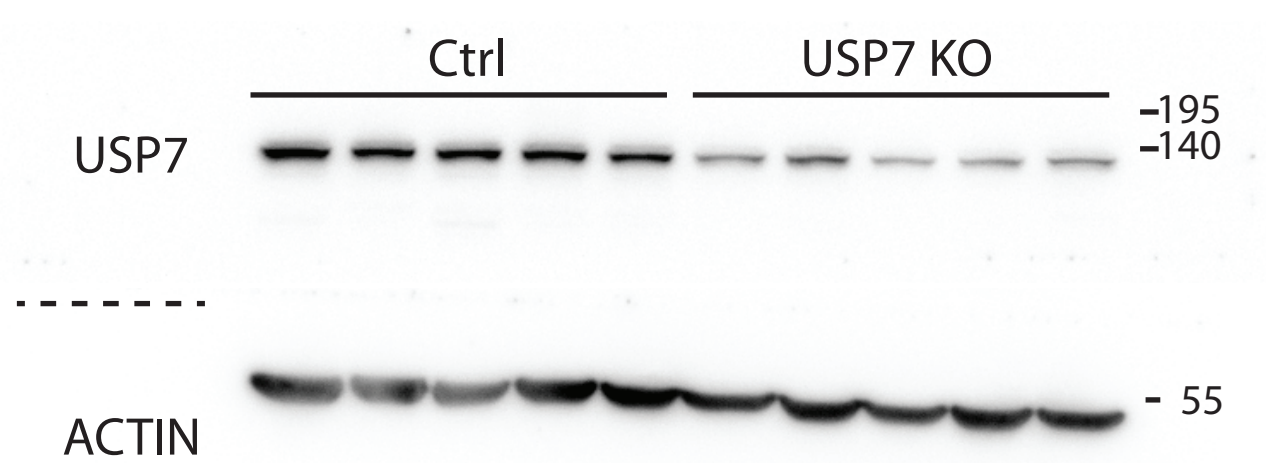

Figure 6C

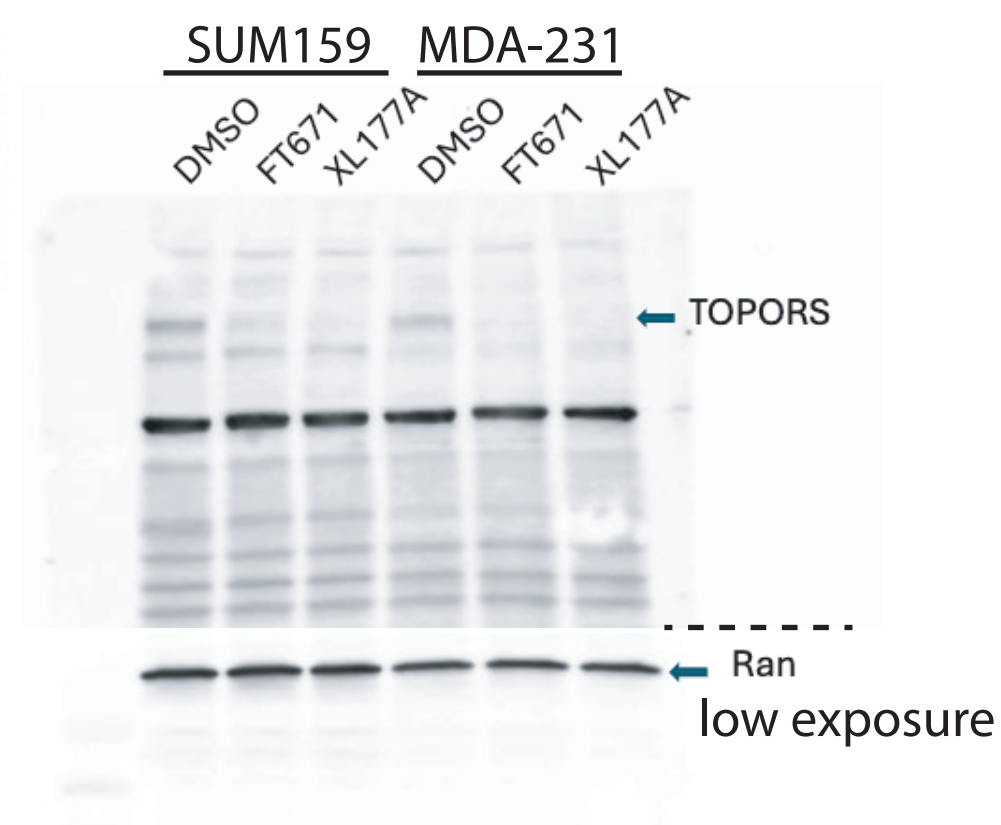

Figure 6D

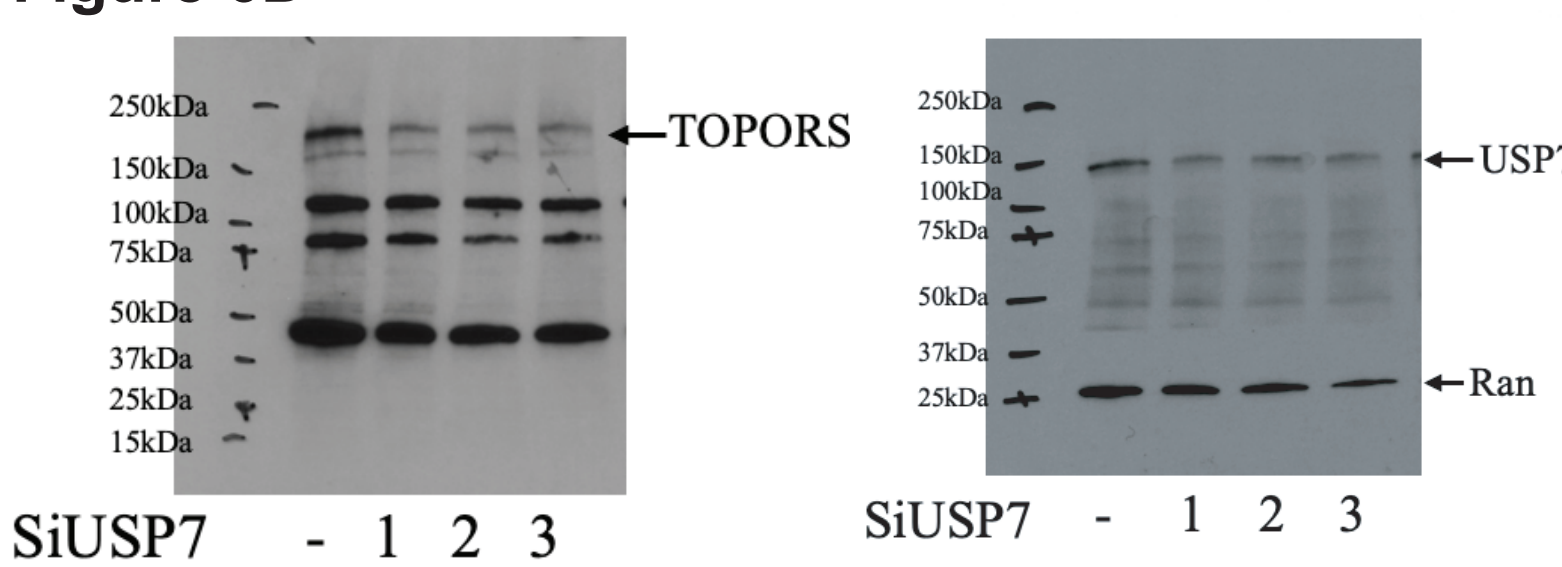

Figure 6E

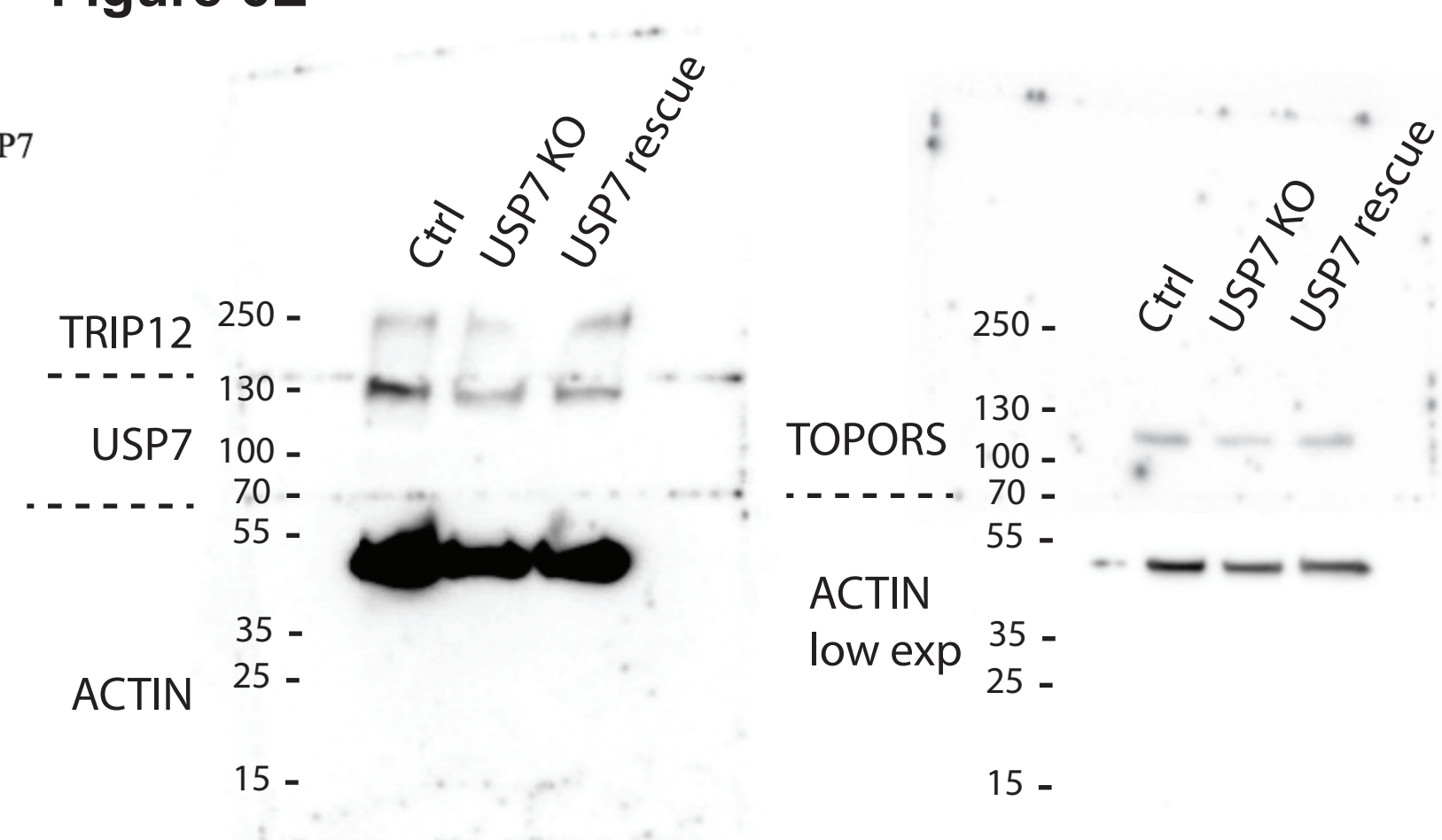

Figure 6F

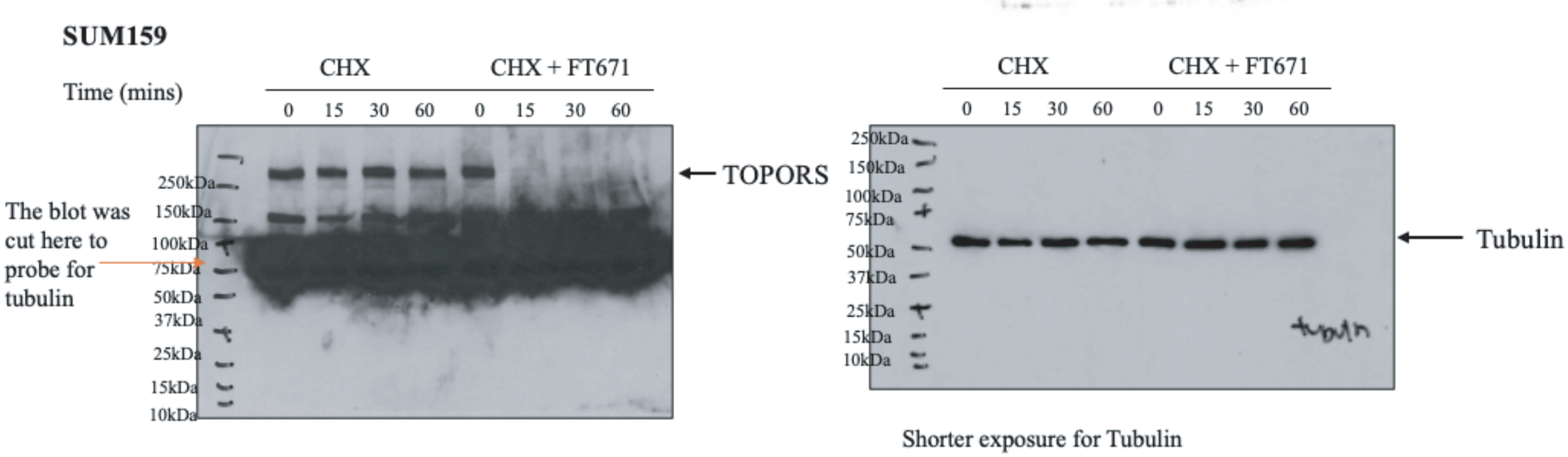

Kim A. et al, Unprocessed immunoblots

Figure 6G

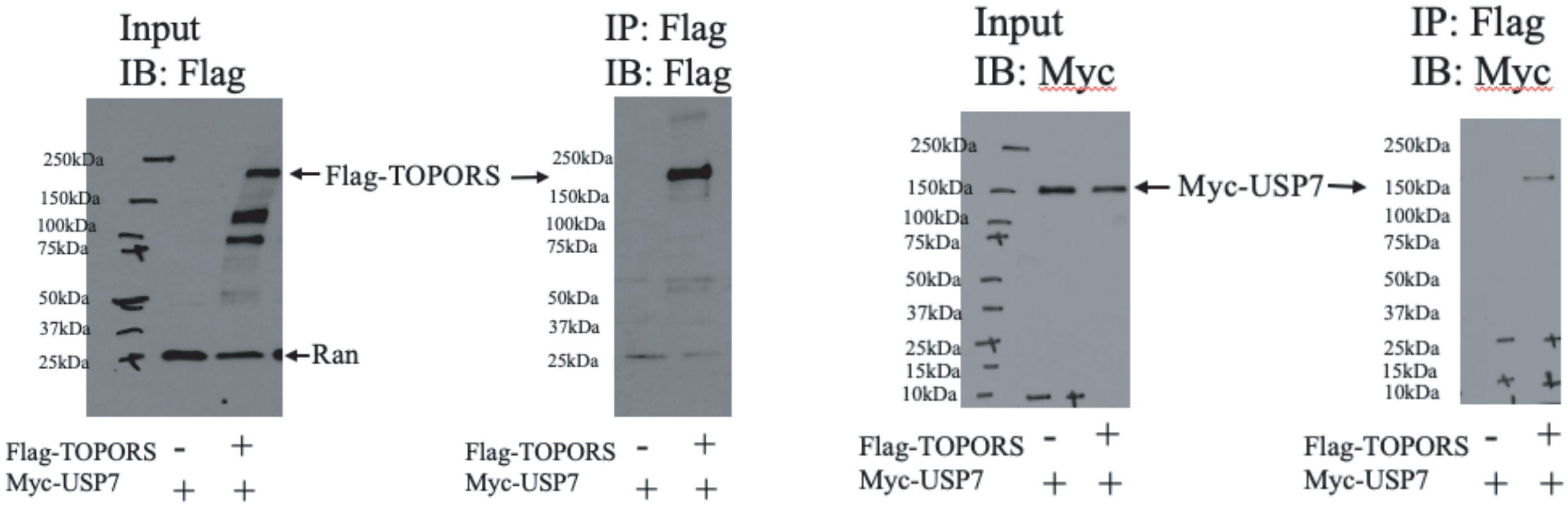

Figure 6H

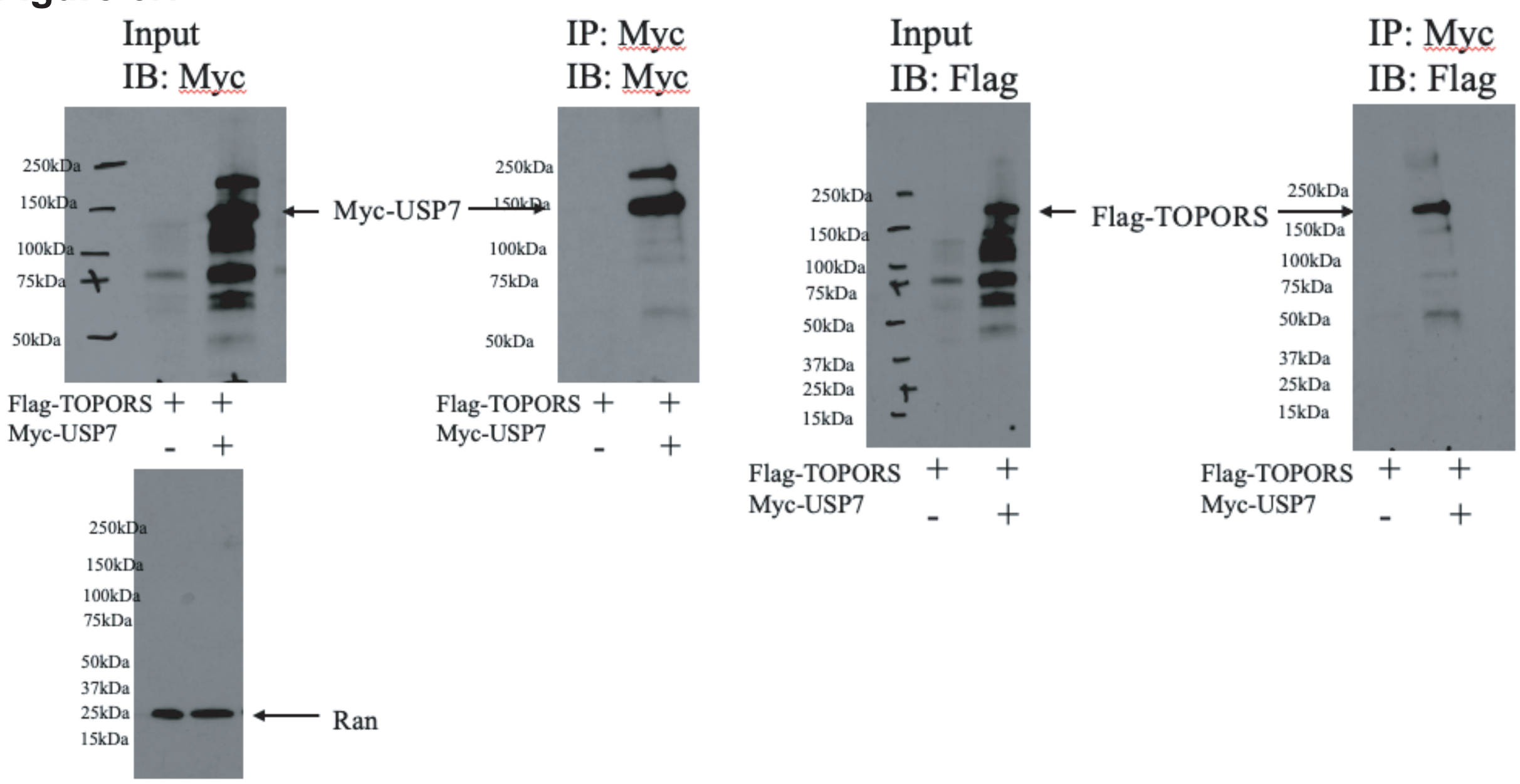

Figure 7D

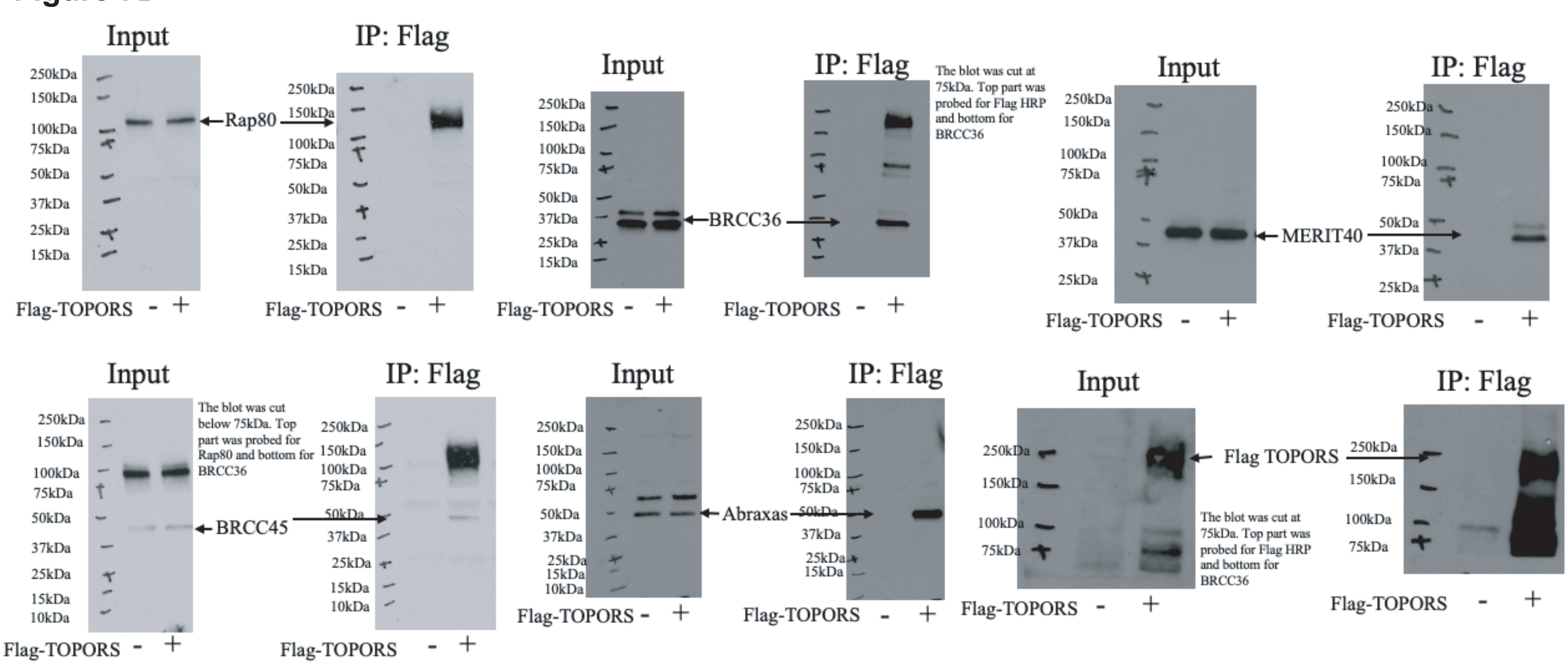

Kim A. et al, Unprocessed immunoblots

Supp. Figure 5B

SUM159

USP7

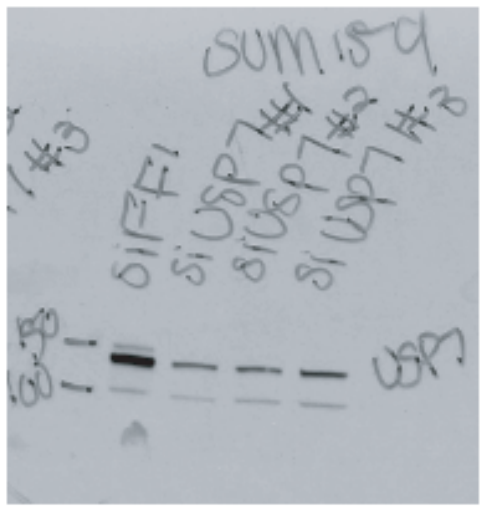

TOPORS

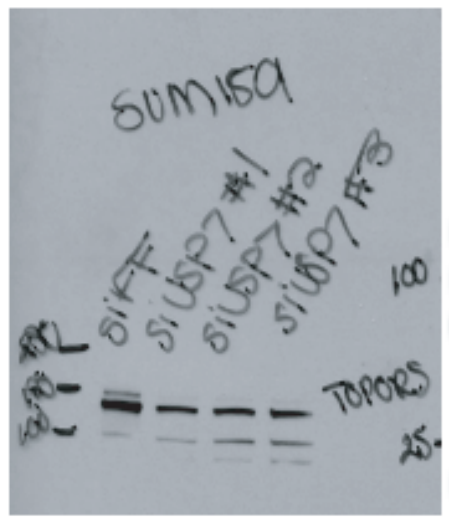

NUCKS1

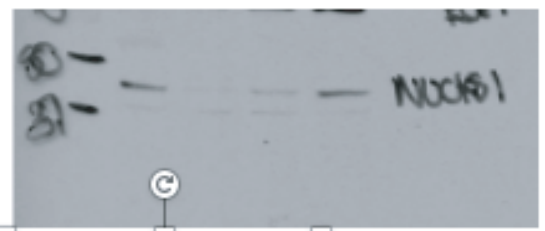

Ran

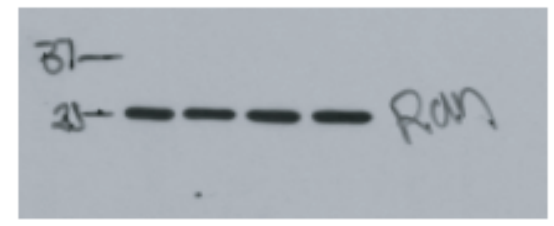

TRIP12

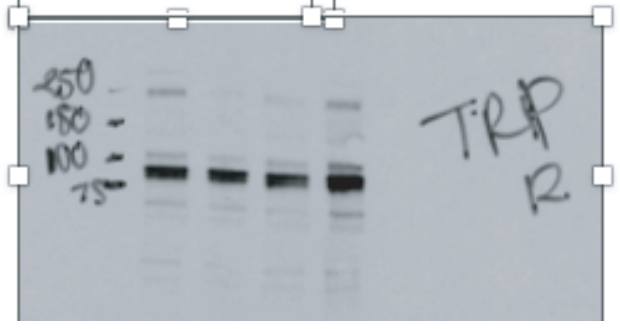

MDA-MB-231

USP7

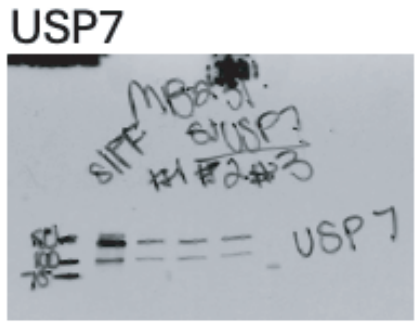

TRIP12

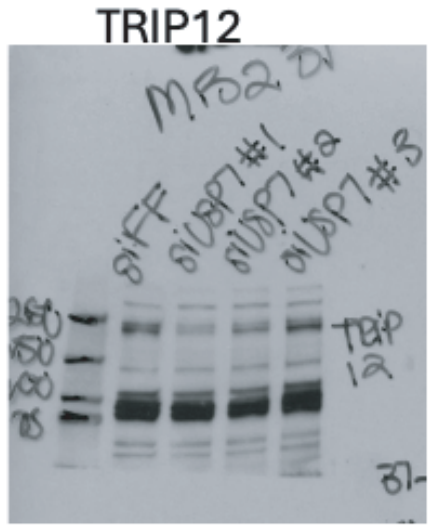

Ran

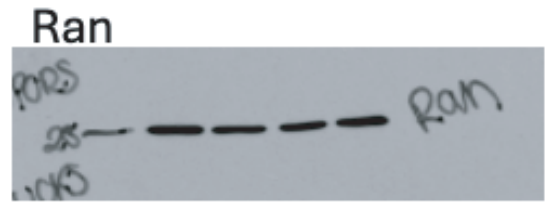

TOPORS

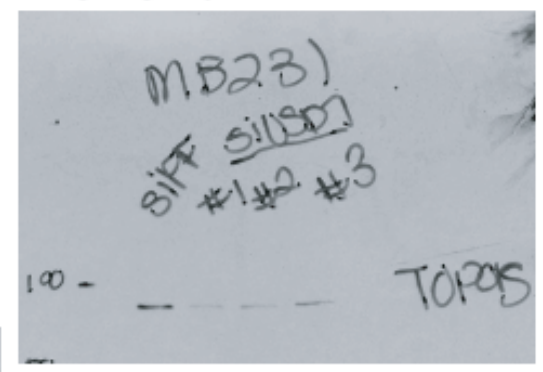

NUCKS1

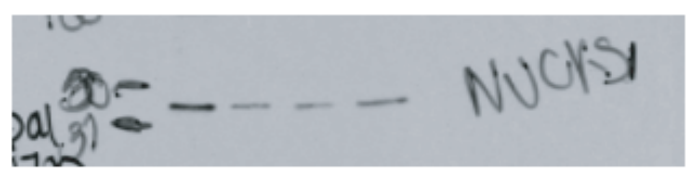

Supp. Figure 5C

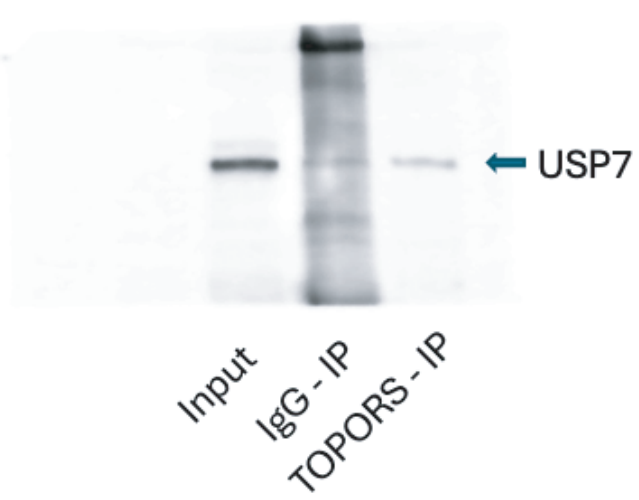

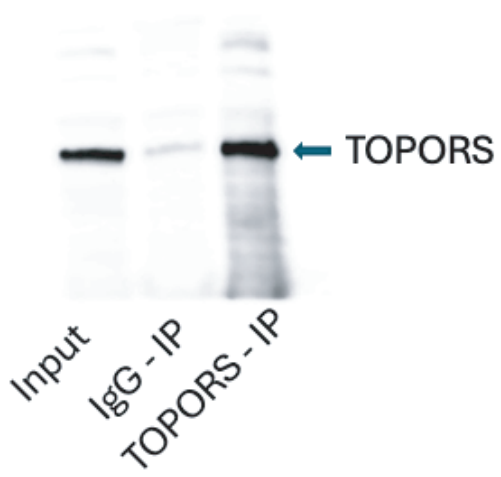

Abraxas

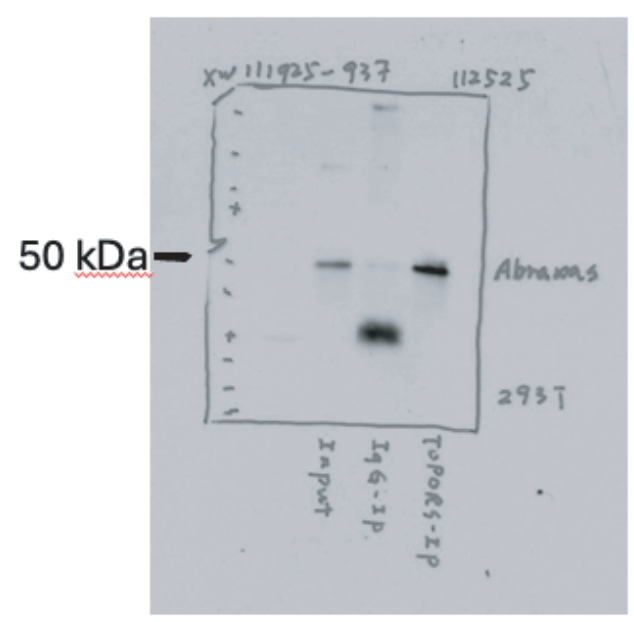

Supp. Figure 6A-C

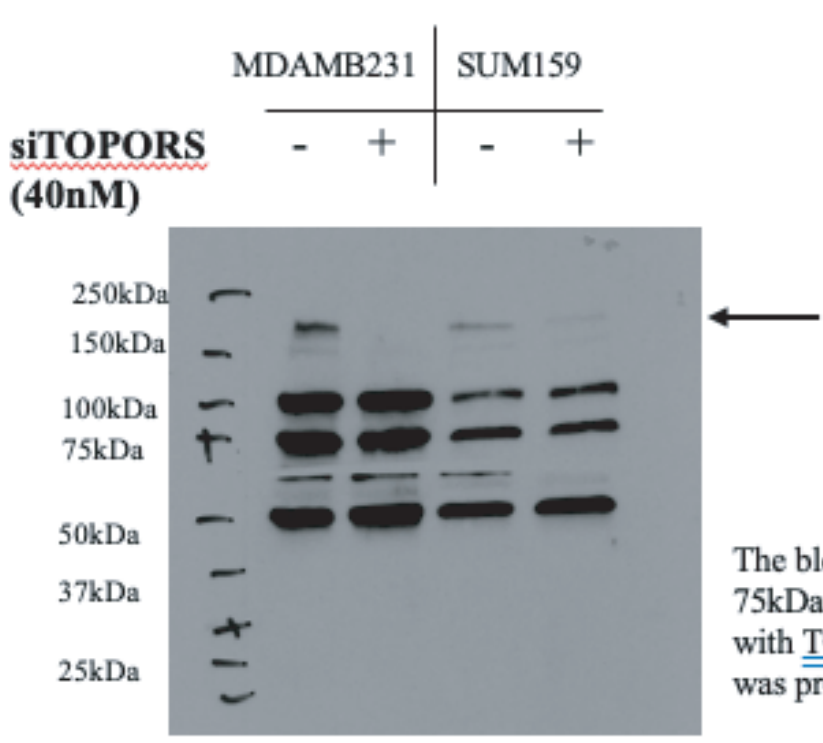

The blot was cut below 75kDa. Top part was probed with TOPORS and bottom was probed with BRCC45

The blot was cut below 75kDa. Top part was probed with Rap80 and bottom was probed with Abraxas

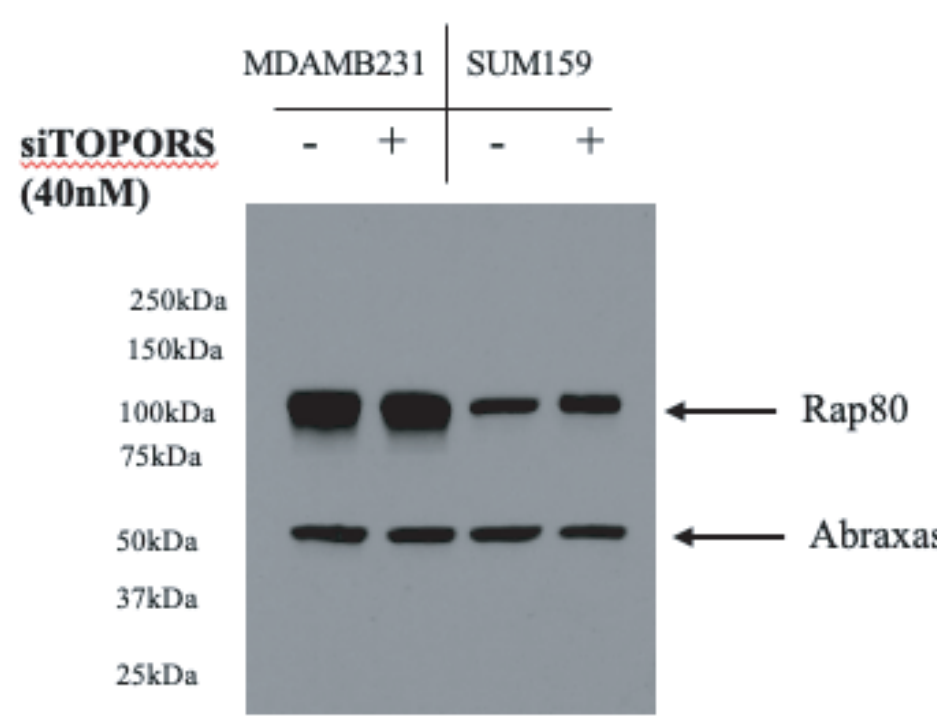

The blot was cut to probe for Ran

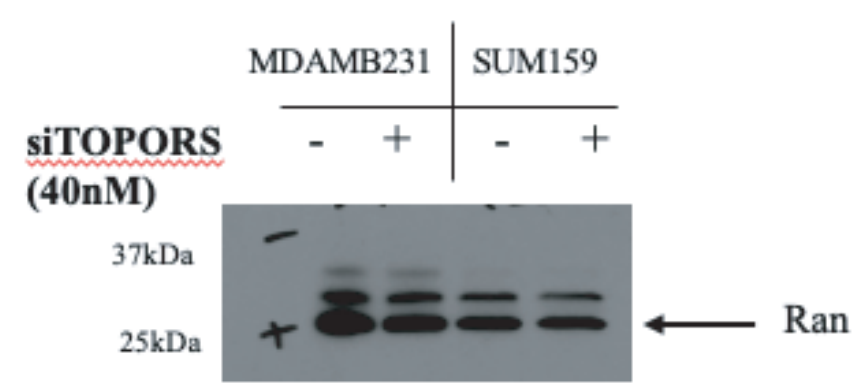

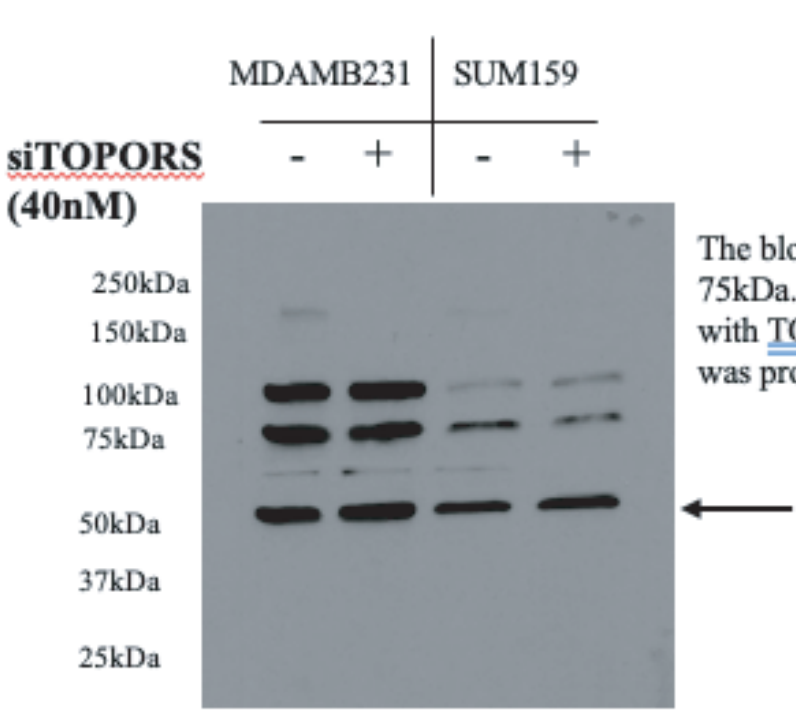

The blot was cut below 75kDa. Top part was probed with TOPORS and bottom was probed with BRCC45

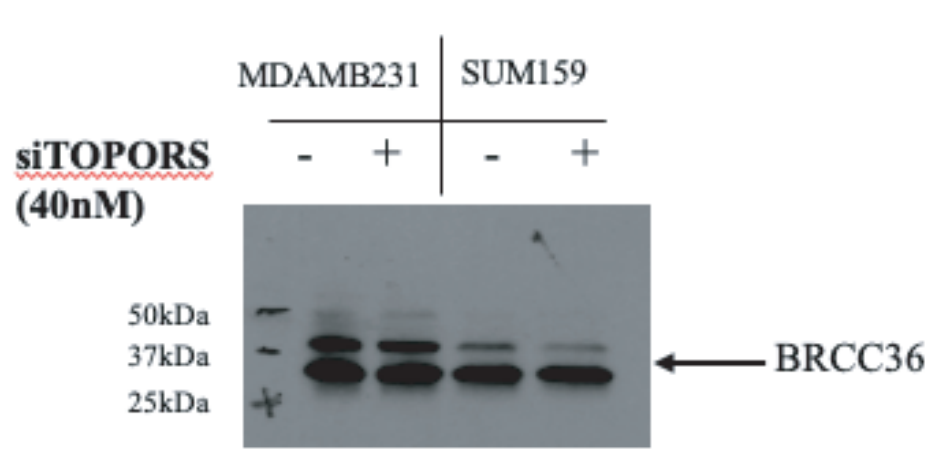

The blot was cut above 50kDa to probe for BRCC36

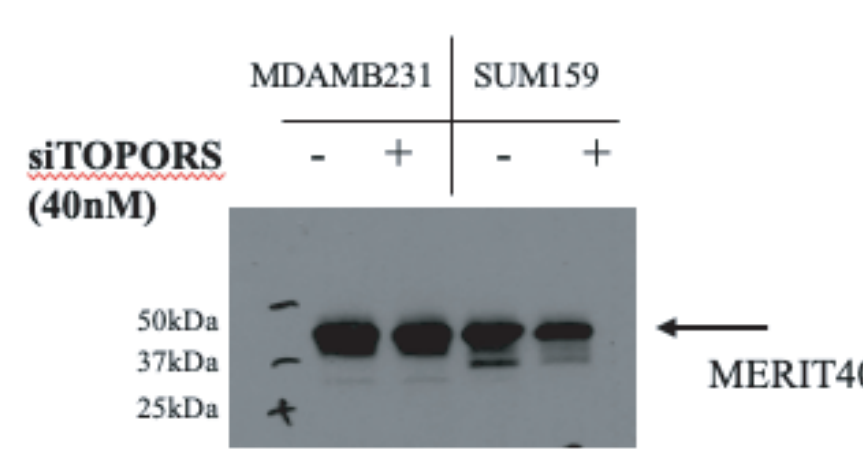

The blot was cut above 50kDa to probe for MERIT40
